# A novel Mediterranean diet-inspired supplement reduces hippocampal amyloid deposits and microglial activation through the modulation of the microbiota gut-brain axis in 5xFAD mice

**DOI:** 10.1080/19490976.2026.2614030

**Published:** 2026-01-13

**Authors:** Emily Connell, Gwénaëlle Le Gall, Simon McArthur, Leonie Lang, Bernadette Breeze, Marrium Liaquat, Matthew G. Pontifex, Saber Sami, Line Pourtau, David Gaudout, Michael Müller, David Vauzour

**Affiliations:** a Norwich Medical School, Faculty of Medicine and Health Sciences, University of East Anglia, Norwich, United Kingdom; b Institute of Dentistry, Faculty of Medicine & Dentistry, Queen Mary University of London, Blizard Institute, London, United Kingdom; c Activ'Inside, Beychac et Caillau, France

**Keywords:** Mediterranean diet, amyloid deposits, microglia, hippocampus, microbial-derived metabolites, gut microbiome, brain

## Abstract

**Background:**

Alzheimer's disease (AD) is projected to increase in prevalence, heightening the need for strategies to alleviate its neuropathological burden. The bioactive constituents of a Mediterranean-style diet are well-recognised for their neuroprotective properties. Due to their capacity to alter the gut microbiome composition, these benefits may involve modulation of the microbiota-gut-brain axis. In this study, we investigated whether a novel supplement enriched with key Mediterranean diet-derived bioactives (Neurosyn240) could reduce amyloid deposition and microglial activation in 5xFAD mice, a transgenic model of AD, through microbiota-mediated mechanisms.

**Methods:**

Male and female 5xFAD transgenic mice (*n* = 16 per sex) were randomly assigned to receive either a standard control diet or a diet supplemented with Neurosyn240 for 12 weeks. Employing a multi-omics approach, gut microbiota composition was profiled using 16S rRNA ampliconsequencing, serum metabolites were quantified via targeted metabolomics, and hippocampal gene expression was analysed through qPCR and RNA sequencing. Neuropathological markers, including amyloid-β deposition and microglial activation, were evaluated using immunofluorescence staining. Statistical analyses were performed using two-way ANOVA to examine the main effects of diet and sex and their interaction.

**Results:**

Neurosyn240 significantly shifted the gut microbiome composition, which was associated with increased circulatory serotonin levels and decreased kynurenine and bile acids (TCA, HDCA, TDCA, CDCA and LCA) concentrations. In the brain, Neurosyn240 consumption led to a significant reduction in hippocampal amyloid deposits and Iba-1 positive microglia (p<0.05), which were associated with decreased LCA and increased serotonin, respectively. Hippocampal RNA sequencing further highlighted the upregulation of genes involved in promoting amyloid beta clearance mechanisms.

**Conclusions:**

Together, these findings highlight novel neuroprotective effects of Neurosyn240 in modulating metabolite-mediated pathways of the microbiota-gut-brain axis, accentuating its therapeutic potential against AD progression.

## 1. Background

Dementia, of which Alzheimer’s disease (AD) is the most common form, is a major and growing public health concern, with an estimated 152.8 million cases predicted worldwide by 2050.^1^ Early therapeutic intervention during the prodromal phase, when neuropathological changes accumulate with minimal or no cognitive symptoms, has been proposed as an effective strategy to slow disease progression and delay the onset of cognitive impairment.^2^ Given this, exploring modifiable factors such as diet has become a promising area of research.

Nutrition is a key influencer of cognitive function and can delay or ameliorate neurodegenerative disease progression.^3-6^ One proposed mechanism linking dietary intake to neuronal health and AD is through the modulation of the extensive bidirectional relationship between the gut microbiome and the brain known as the microbiota-gut-brain axis.^7-9^ Indeed, microbial fermentation of dietary bioactives in the gut produces numerous soluble metabolites that have been shown to exert anti-inflammatory and protective effects upon the blood-brain barrier (BBB) and the microglia, key actors in neurodegenerative diseases.^10^ For example, secondary bile acids produced by gut microbes can circulate through the blood and cross the BBB to activate TGR5 or FXR to inhibit microglial activity for an anti-inflammatory effect via attenuation of ERK pathway.^11^ Importantly, the BBB and microglia are known to be impaired early in the pathogenesis of AD, hence we hypothesize that strategies to promote their function may have considerable therapeutic benefit.

The Mediterranean diet, composed of a high intake of fruits, vegetables, olive oil, whole grains and unsaturated fatty acids, moderate intake of fish and restricted consumption of red meats,^3^ has been reported to improve cognitive function^12-14^ and slow age-related cognitive decline.^15^^,^^16^ Potential mechanisms underlying the protective effects of the Mediterranean diet include a combination of anti-inflammatory, antioxidant, vasculoprotective and microbiome-modulating properties.^17-19^ Given the growing evidence that the Mediterranean diet can influence gut microbiome composition,^18^ we hypothesized these effects may be mediated through the response of the microbiota-gut-brain axis.

Here, we explore the effects of consumption of a novel blend of bioactives commonly found in the Mediterranean diet, named 'Neurosyn240’ thereafter, on the microbiota-gut-brain axis in male and female 5xFAD mice, determining its potential as an early preventative dietary intervention to delay AD progression. 5xFAD is an early-onset mouse model of AD displaying amyloid pathology and hippocampal-dependent cognitive deficits from 6 to 13 months of age.^20^^,^^21^ Our study focuses on the prodromal stage of AD in this model, a critical period before cognitive symptoms appear, but when metabolic changes may influence future cognitive decline. This stage represents a key window for implementing nutritional strategies to slow disease progression. Beneficial effects of the Mediterranean diet have been shown to be sexually dimorphic, with greater protection against AD observed in women.^22^^,^^23^ However, most characterisation studies using 5xFAD mice overlook sex-specific differences^24^ or include only one sex,^25^ which might have significant consequences when translating the findings to humans. To address these shortcomings, the current study assessed changes in the microbiota-gut-brain axis and neuropathology in the prodromal stages of decline in both male and female 5xFAD mice (up to 5 months of age).^26^

## 2. Methods

### 2.1. Experimental procedure

5xFAD mice with a C57Bl/6 background overexpress human amyloid precursor protein (APP) with three FAD mutations [the Swedish (K670N, M671L), Florida (I716V), and London (V7171) mutations] and human PSEN1 with two FAD mutations (M146L and L286V).^27^ The 5xFAD mouse model of AD displays intraneuronal amyloid beta (Aβ) from 6 weeks of age, neuronal loss in the subiculum and cortical layer V at approximately 39 weeks^24^ and hippocampal-dependent cognitive deficits from 26 to 56 weeks of age.^20^^,^^21^ 16 males and 16 females 5xFAD mice (6 weeks old) sourced from Jackson Laboratories (Bar Harbor, US) were maintained in individually ventilated cages (*n* = 4 per cage), in a controlled environment (21 ± 2 °C; 12 h light/dark cycle; light from 7:00 AM) and fed *ad libitum* on a standard diet (RM3-P; Special Diet Services (SDS, Horley UK) for 2 weeks ensuring normal development and stabilisation of the microbiota.^28^ After this time, mice were transferred onto one of two diets, namely control (AIN93-M) or a Neurosyn240 supplemented diet for 12 weeks. The Neurosyn240 diet comprises of the background control diet (AIN93-M) supplemented with 2,157 mg/kg diet of Neurosyn240 (Activ’Inside, Beychac-et-Caillau, France). Neurosyn240 is a proprietary (patent pending) standardized blend of Memophenol™, a unique formula of French Grape (*Vitis vinifera L*.) and North-American Wild Blueberry (*Vaccinium angustifolium A*.) extract (patent WO/2017/072219), saffron extract (patent WO/2018/020013), green tea extract, olive leaf extract, trans-resveratrol, zinc, vitamins B5, B9, B12, C, D3 & E (comprising 15% of vitamins and mineral reference value), polyphenols (mainly flavan-3-ol monomers ≥10% and resveratrol ≥1%) and crocin carotenoids (mainly trans-4-galloyl-gallate, trans-3-galloyl-gallocatechin, cis-4-galloyl-gallate, trans-2-galloyl) ≥500 ppm as previously described.^29^ The dietary fiber content of this mix is at trace amounts and therefore is unlikely to exert an effect upon either the gut microbes or host systems. Diets were prepared by Research Diets Inc. (New Brunswick, USA) to comply with animal nutrition requirements. At the end of the experiments, 5-month-old animals were anaesthetized with a mixture of isoflurane (1.5%) in nitrous oxide (70%) and oxygen (30%) and transcardially perfused with ice-cold saline containing 10 UI/ml heparin (Sigma-Aldrich, UK). Cardiac blood was allowed to clot for 30 mins on ice before sera were isolated via centrifugation at 4,000 x g for 10 min. Brains were rapidly removed, halved, snap frozen and stored at −80 °C until biochemical analysis. Additionally, caeca were removed, weighed and contents were extracted.

### 2.2. Behavioral assessment

All behavioral tests were performed at the experimental endpoint after the 12-week intervention, with mice remaining on the control or Neurosyn240 diet during the testing period. Baseline behavioral testing was not conducted, as prior exposure to the tasks can lead to learning effects, which may confound the interpretation of treatment-related improvements.^30^ To ensure that observed differences reflect the impact of the intervention rather than task familiarity, animals were naïve to the behavioral tests at the time of assessment. Prior to commencing, a visual placing test was performed on each animal to ensure animals were not visually impaired.^31^ All behavioral tests were analyzed using the Ethovision software (Tracksys Ltd, Nottingham, UK).

The Open Field (OF) task, a measure of anxiety-related behavior^32^, was conducted as described previously.^33^ Animals were individually placed within the (50 cm × 50 cm × 50 cm cubed) arena illuminated with dim lighting (100 lux) and were allowed to freely move for 10 minutes. Mice were tracked using Ethovision software which determined travel distance, velocity and time spent in the center/periphery of the maze respectively.

The novel object recognition (NOR) task, a measure of recognition memory, was conducted as previously described.^34^^,^^35^ All experiments were carried out under dim lighting conditions (100 lux). Briefly, on day 1 (habituation), mice were placed into an empty arena (50 cm × 50 cm × 50 cm) for 10 minutes. On day 2, animals were conditioned to two identical objects for 10 minutes. Half of the animals were conditioned with two identical golf balls and the other half with two green plastic toy bricks (4x4x6 cm). Following an inter-trial interval of one hour, mice were placed back within the testing arena now containing one familiar object and one novel object, with the position of the novel object (left or right) being randomized between each mouse and group tested. Mice conditioned with the golf balls had the plastic toy brick placed as the novel object and vice versa. Videos were analyzed for a 5-minute period. If the mice did not accumulate a total of 8 seconds of object exploration within this time, the analysis continued for up to 10 minutes or until 8 seconds of exploration was reached. Mice not achieving 8 seconds of exploration were excluded from the analysis.^36^ Discrimination index (DI) was calculated as follows DI = (TN − TF)/(TN + TF), where TN is the time spent exploring the novel object and TF is the time spent exploring the familiar object.

The Y-maze spontaneous alternation test, a measure of spatial working memory, was conducted as previously described.^37^ Ethovision software analyzed each animal for 7 minutes recording zone transitioning and locomotor activity. Spontaneous alternation, defined as the tendency of rodents to explore a new arm of the maze rather than returning to one previously visited, reflecting their working memory, was calculated using the formula: (Number of alternations/Total Arm entries - 2) x 100).

The Barnes maze, as previously described,^38^ was performed with slight modifications to assess spatial retrieval memory. Briefly, the maze consisted of a brightly illuminated (800 lux lighting) circular platform (92 cm diameter), with 20 evenly distributed holes located around the circumference and visual cues (4 simple shapes) placed at the periphery. The experiment was conducted over a 5-day period. On day 1 (habituation) animals were placed into the center of the maze for 2 minutes and were able to explore freely. If the animal did not find the escape box within this time frame the animal was guided to it after which they remained in the box for a further 2 minutes. Following habituation each mouse was tested/trained on their ability to locate the escape box on days 1–4 with three trials per day. On day 5, a probe test was conducted, the maze was rotated 90°, the escape box was removed, and mice were placed in the center of the maze in which they were free to navigate for 1 minute. Percentage time in the correct quadrant was determined using Ethovision software.

### 2.3. RNA isolation and mRNA sequencing

Microbial DNA was isolated from approximately 50 mg of cecal contents using a FastDNA SPIN Kit for Soil (MP Biomedicals). Samples were quantified using a nanodrop (ND-1000 spectrophotometer) and quality assessment was performed by agarose gel electrophoresis to detect DNA integrity, purity, fragment size and concentration. The 16 S rRNA amplicon sequencing of the V3–V4 hypervariable region was performed with an Illumina NovaSeq 6000 PE250 using a paired-end 150 bp sequencing strategy. Sequence analysis was performed by Uparse software (Uparse v7.0.1001) 37,^39^ using all the effective tags. Sequences with ≥97% similarity were assigned to the same operational taxonomic units (OTUs). A representative sequence for each OTU was screened for further annotation. Each sequence was analyzed using Mothur software against the SSUrRNA section of the SILVA database.^40^ OTUs abundance information was normalized using a standard sequence number corresponding to the sample with the least sequences. Alpha diversity was assessed using Chao1 and Shannon H diversity indices whilst beta diversity was assessed using Bray–Curtis. Statistical significance was determined by Wilcoxon rank-sum test (Mann–Whitney-U) or a permutational multivariate analysis of variance (PERMANOVA).

### 2.4. LC-MS/MS

Liquid chromatography with tandem mass spectrometry (LC-MS/MS) was conducted to gain insight into possible shifts in the production of bioactive serum metabolites and targeted metabolites previously associated with cognitive health, including bile acids, tryptophan, trimethylamine *N*-oxide (TMAO), *p*-cresol and its derivatives.^41^ Serum samples (80 µL) were diluted with methanol at a ratio of 1:10 (*v/v*) and placed on dry ice for 10 min. Samples were then centrifuged (5 min, 16,000x g at room temp), supernatants filtered using a 0.45 µM PTFE syringe filter and evaporated to dryness using a Savant™ SpeedVac™ High-Capacity Concentrator (Thermofisher, UK). Dried samples were resuspended in either 50 µL of methanol with the addition of 15 µL of lithocholic acid-d4 and cholic acid-d4 at 50 µg/mL for the detection of bile acids, 50 µL water with TMA-d9 *N*-oxide, TMA ^13^C ^15^*N* hydrochloride at 50 µg/mL for the detection of TMAO/TMA/choline or 50 µL water with 15 µL of L-methionine-3, 3, 4, 4 d4 and *p*-toluenesulfonic acid at 50 µg/mL for the detection of tryptophan and *p*-cresol metabolites respectively. All internal standards were supplied from Thermofisher, UK. Samples were analyzed using Waters Acquity UPLC system and Xevo TQ-S Cronos mass spectrometer with MassLynx 4.1 software. For the detection of bile acids, the electrospray ionization (ESI) operated in negative mode and chromatographic separations were performed with a Supelco Ascentis Express C18 column (150 × 4.6 mm, 2.7 µM). A binary gradient of eluent A (10 mM ammonium acetate, 0.1% formic acid, water) and eluent B (10 mM ammonium acetate, 0.1% formic acid, methanol) ran at a constant rate of 0.6 mL/min. For the quantification of TMAO, TMA and choline, ESI operated in positive mode. Chromatographic separation was achieved using an Ethylene Bridged Hybrid (BEH) Amide (150 × 2.1 mm, 1.7 µM) and 0.5 mL/min using eluent A (10 mM ammonium formate, 0.2% formic acid, 50% acetonitrile) and eluent B (10 mM ammonium formate, 0.2% formic acid, 95% acetonitrile). For the detection of tryptophan and *p*-cresol related metabolites, ESI operated in positive mode using an ACQUITY UPLC BEH C18 1.7 µM (2.1 x 50mm) column. A binary gradient of eluent A (0.1% formic acid, water) and eluent B (0.1% formic acid, methanol) held a constant flow rate of 0.3 mL/min. Chromatogram peak analysis was performed by the accompanying Waters® TargetLynx ™ application manager. Gradients for all three methods are described elsewhere.^42^

### 2.5. Immunofluorescent staining

Coronal brain cryosections (20 µm) were processed from snap-frozen hemi-brain samples. Cryosections were mounted onto glass slides before being fixed in 4% formaldehyde in 0.1 M PBS for 15 minutes. To detect amyloid deposits, tissue sections were incubated in 1% thioflavin S aqueous solution (Sigma-Aldrich) for 8 minutes. Sections were further washed twice in 80% ethanol, once in 95% ethanol and thrice in distilled water prior to mounting with Mowiol mounting medium and storage at −20 °C.

To detect the microglial marker protein, Iba-1, sections were incubated for 30 min at room temperature in 10% normal goat serum (NGS) containing 0.05% Triton X-100 to permeabilise cells and block nonspecific secondary antibody binding. Sections were then incubated for 1 hour at room temperature in a rabbit anti-mouse Iba-1 polyclonal antibody (Synaptic Systems) diluted 1/1000 in 1% NGS containing 0.05% Triton X-100. Sections were further washed in 1% NGS and incubated for 1 hour with an AF555-conjugated goat anti-rabbit IgG secondary antibody (diluted 1/500) and washed twice in PBS. 4',6-diamidino-2-phenylindole (DAPI) was used as a counterstain to visualize cell nuclei. Tissue samples were mounted in an aqueous medium, Mowiol, dried and stored at −20 °C.

Images of the hippocampus were collected for the analysis of amyloid deposit pathology and Iba-1 positive microglia using a Direct Fluorescence Leica Ctr 5000 Microscope at x10 magnification. Image analysis was performed manually using Fiji software (NIH, Washington DC, USA). The area of the region of interest (ROI) was outlined manually. Counts of thioflavin S-positive deposits represent the hippocampal region. Iba-1 positive microglia were counted within a randomly selected 200 µm^2^ ROI in the hippocampus.

### 2.6. RNA isolation and RNA sequencing

Total RNA was isolated from hippocampi (*n* = 3 randomly selected per group and sex) using the Qiazol reagent (Qiagen, UK). Messenger RNA was then purified from total RNA using poly-T oligo-attached magnetic beads. After fragmentation, the first strand cDNA was synthesized using random hexamer primers, followed by the second strand cDNA synthesis using either dUTP for directional library or dTTP for non-directional library.^43^ The libraries were checked with Qubit and real-time PCR for quantification and bioanalyser for size distribution detection. Quantified libraries were pooled and sequenced on Illumina NovaSeq 6000 platform using a paired-end 150 bp, according to effective library concentration and data amount. Raw data (raw reads) of FASTQ format were first processed through in-house perl scripts. The index of the reference genome was built using Hisat2 v2.0.5^44^ and paired-end clean 1 reads were aligned to the reference genome using Hisat2 v2.0.5. FeatureCounts v1.5.0-p3 was used to count the reads numbers mapped to each gene.^45^ FPKM of each gene was calculated based on the length of the gene and reads count mapped to this gene.

Data was analyzed using the DESeq2 package in R (version 4.3.2) to investigate the effects of diet and sex on gene expression. Raw count data were filtered to remove low-abundance genes, retaining those with a minimum count of 5 in at least 15% of the samples. Genes with less than 15% variance were excluded. A generalized linear model (GLM) assessed the main effects of diet and sex. Differential expression was tested for each factor, which captures differential responses to diet between sexes. *P*-values were adjusted using the Benjamini-Hochberg method to control the false discovery rate (FDR). Enrichr was used to perform Gene Ontology (GO) analysis of differentially expressed genes (DEGs).^46^^,^^47^

### 2.7. qRT-PCR

RNA was prepared as outlined above. Two μg of total RNA was treated with DNase I (Invitrogen, UK) and used for cDNA synthesis using Invitrogen™ Oligo (dT) primers and M-MMLV reverse transcriptase. Quantitative real-time PCR (qRT-PCR) reactions were performed using SYBR green detection technology on the Applied Biosystems QuantStudio 5 Real-Time PCR system (Life Technologies). Results are presented as relative fold change in Neurosyn240 females and males compared with sex-matched control groups. Values were scaled to the average across all samples for each target gene and normalized to the mean of the reference genes TATA-box binding protein (*Tbp*) and hypoxanthine phosphoribosyltransferase 1 (*Hprt1*). The primer sequences are given in Supplementary Table S1.

### 2.8. Statistical analysis

Data analysis was performed in GraphPad Prism version 9.5.1 (GraphPad Software, CA, USA). All values are presented as means ± standard error of the means (SEM) unless otherwise stated. After identifying outliers using the ROUT method (q = 1%), data were checked for normality using the Shapiro–Wilk test. Analyzes were conducted using two-way ANOVA with Tukey *post-hoc* analysis when appropriate to correct for multiple comparisons. Weekly body weights for all mice were analyzed using repeated measures two-way ANOVA to assess the effects of diet, sex, and time, as well as their interactions on body weight. Partial Least Squares Discriminant Analysis (PLS-DA) was employed to illustrate the clustering of different metabolites across groups using Metaboanalyst 5.0.^48^ Dendrogram and heatmaps were created using Spearman's rank correlation for distance measurement and Ward's method for hierarchical clustering. Procrustes analysis, investigating the congruence of the metabolomics and microbiome data, was conducted using M2IA.^49^ All other correlation analyzes were conducted using Spearman's rank-order correlation analysis. *p* values of less than 0.05 were considered statistically significant.

## 3. Results

### 3.1. Neurosyn240 supplementation does not affect body weight or food intake in both males and females

**Figure 1.**
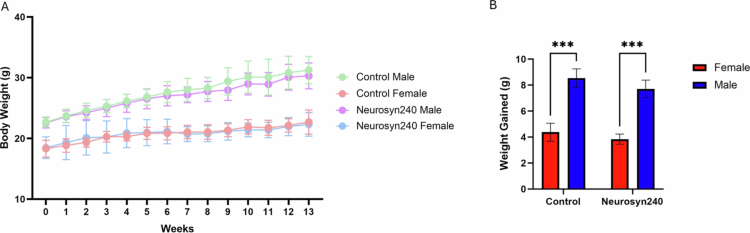
Supplementation of the Neurosyn240 diet for 12 weeks showed no significant difference in body weight to the control diet in both males and females. (A) weekly average body weight of males and females on the Neurosyn240 and control diet over 13 weeks. (B) Total weight gained by males and females on the control and Neurosyn240 diet over the course of the 12-week intervention. Error bars represent SEM. *** = *p* < 0.001.

Whilst body weight increased over the 12-week intervention for both males and females, animals receiving Neurosyn240 supplementation did not significantly differ from the control group of the same sex (Figure 1A). A main effect of sex was detectable upon body weight (F(1, 28) = 40.4; *p* < 0.0001), but neither diet (F(1, 28) = 1.16; *p* = 0.29) nor the interaction between sex and diet (F(1, 28) = 0.05; *p* = 0.82) reached statistical significance (Figure 1B). There were also no significant differences in food intake throughout the study with both males and females consuming on average 2.9 ± 0.3g per animal, per day, providing an average of 208 ± 21 mg/kg BW/day of Neurosyn240. Using allometric scaling based on body surface area,^50^ this dose equates to 1.18 ± 0.1 g/day human equivalent dose for a person of 70 kg.

### 3.2. The Neurosyn240 containing diet does not significantly modulate cognitive decline

**Figure 2.**
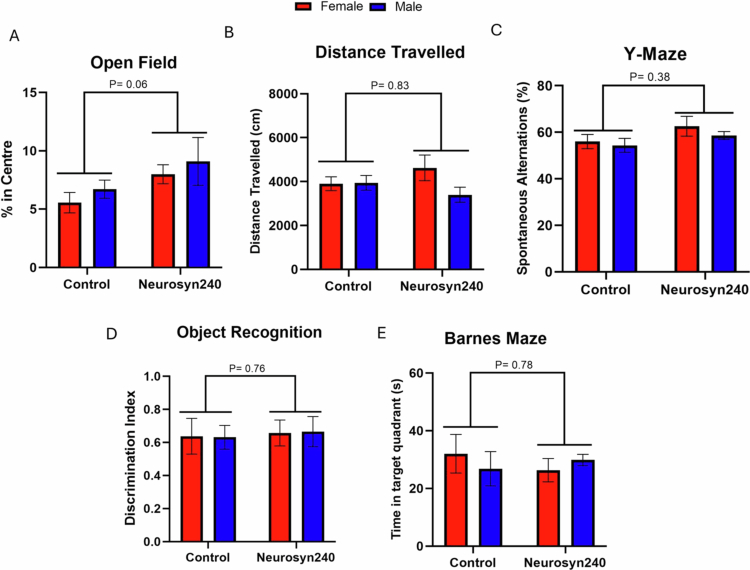
Neurosyn240 diet did not significantly alter hippocampal-dependent behavioral measures. (A) Percentage of time spent in the center of the open field area in males and females consuming control and Neurosyn240 diet. (B) Distance traveled by mice during open field assessment. (C) Performance of mice on Y-maze measured by spontaneous alternation. (D) Performance of mice on novel object recognition assessment as measured by the discrimination index. (E) Percentage of time spent in the target quadrant on day 5 of the Barnes maze test. All *p*-values represent the main effect of diet on behavioral assessments.

After 12 weeks of dietary intervention, cognitive performance was assessed in 5-month-old 5xFAD mice. There was a near significant effect of diet on time spent in the center of the open field test as a measure of anxiety (F(1, 27) = 4.09; *p* = 0.06; Figure 2A), but no significant effect of distance traveled in the open field (F(1,27) = 0.04; *p* = 0.83; Figure 2B), or performance on the Y-maze (F(1,27) = 2.9; *p* = 0.38; Figure 2C), novel object recognition tests (F(1,27) = 0.097; *p* = 0.76; Figure 2D) or Barnes maze (F(1,27) = 0.078; *p* = 0.78; Figure 2E). No significant effects of sex were seen in the open field assessment on time spent in the center (F(1,27) = 0.90; *p* = 0.35) or distance traveled in the open field (F(1,27) = 2; *p* = 0.16), as well as Y-maze (F(1,27) = 0.79; *p* = 0.38), novel object recognition (F(1,27) = 0.0001; *p* = 0.99) and Barnes maze (F(1,27) = 0.028; *p* = 0.87). No significant interactions between diet and sex were detected in the behavioral measures.

### 3.3. Neurosyn240 diet significantly modulates the intestinal microbiota

**Figure 3.**
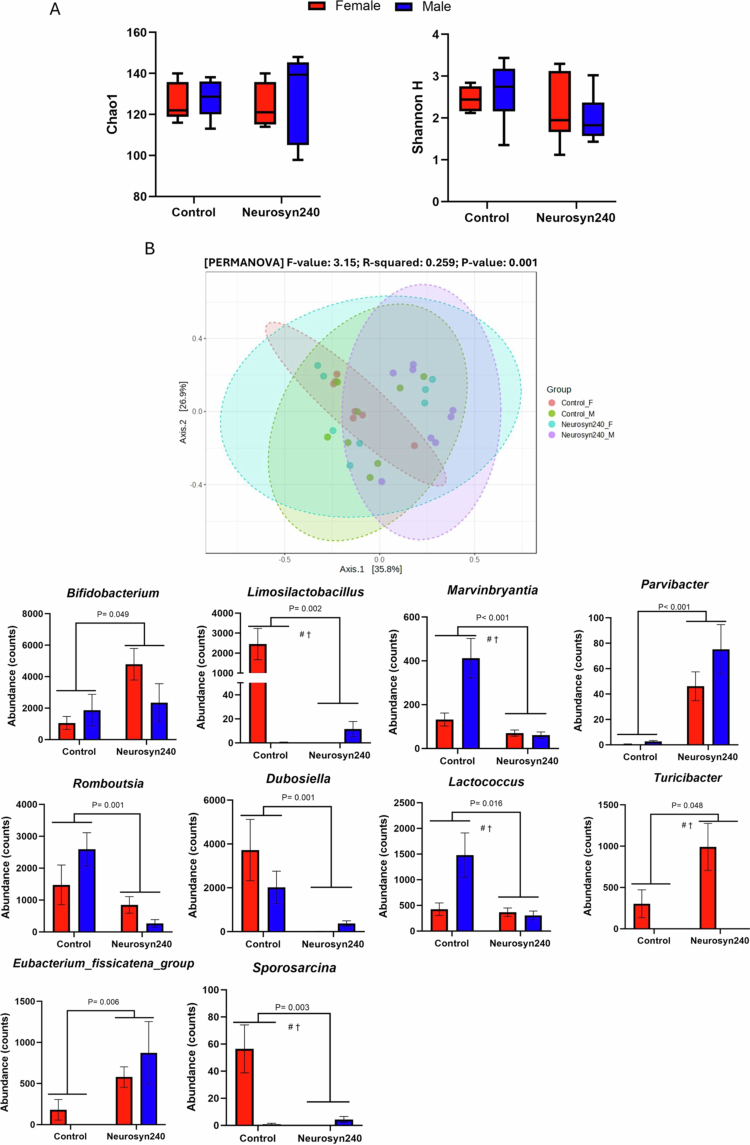
Neurosyn240 diet altered beta, but not alpha diversity of the gut microbiome. (A) Alpha diversity as measured by Chao1 and Shannon H index was not significantly modulated by the main effect of diet, sex or their interaction. (B) Beta diversity as measured by Bray-Curtis; *p*-value generated from PERMANOVA. (C) Abundance of microbiome genera significantly affected by the main effect of diet (*p* < 0.05). *P*-value indicates the statistical significance of the main effect of diet. # denotes a significant main effect of sex. † indicates a significant interaction between sex and diet (*p* < 0.05).

The impact of Neurosyn240 on microbial composition was investigated by 16S rRNA amplicon sequencing. Alpha diversity, as measured by the Chao1 index, was not significantly affected by sex (F(1,27) = 0.21; *p* = 0.65) or diet (F(1,27) = 0.53; *p* = 0.47), nor was there an interaction between sex and diet (F(1,27) = 0.21; *p* = 0.65). Shannon H index displayed near significant effects of sex (F(1,27) = 0.03; *p* = 0.06) and diet (F(1,27) = 3.87; *p* = 0.06) but no significant interaction term (F(1,27) = 0.75; *p* = 0.39) (Figure 3A). Principal coordinates of analysis (PCoA) highlighted significant differences in beta diversity metrics (PERMANOVA F = 3.15; R² = 0.26; *p* = 0.001) between males and females in both control and Neurosyn240 groups (Figure 3B). Pairwise comparisons suggested both males (F = 4.39; R² = 0.24; FDR q = 0.018) and females (F = 1.98; R² = 0.13; FDR q = 0.09) had significant differences in beta diversity with the consumption of Neurosyn240 (FDR q < 0.1) (Supplementary Table S2). At the genus level, linear discrimination analysis (LDA) effect size (LEfSe) revealed significant differences in 16 genera between groups (FDR q < 0.05; Supplementary Figure S1). Of these bacteria, a main effect of diet was detected in *Lactococcus* (F(1,27) = 6.67; *p* = 0.016), *Limosilactobacillus* (F(1,27) = 12.12; *p* = 0.002), *Marvinbryantia* (F(1,27) = 17.22; *p* < 0.001), *Parvibacter* (F(1,27) = 25.52; *p* < 0.001), *Romboutsia* (F(1,27) = 12.69; *p* = 0.001), *Sporosarcina* (F(1,27) = 10.88; *p* = 0.003), *Turicibacter* (F(1,27) = 4.30; *p* = 0.048), *Eubacterium_fissicatena_group* (F(1,27) = 8.77; *p* = 0.006), *Bifidobacterium* (F(1,27) = 4.26; *p* = 0.049) and *Dubosiella* (F(1,27) = 13.14; *p* = 0.001) (Figure 3C; Supplementary Table S3 for all genera abundances). Eight genera were significantly influenced by the main effect of sex, including *Lactococcus* (F(1,27) = 4.28; *p* = 0.048), *Limosilactobacillus* (F(1,27) = 12.12; *p* = 0.0017), *Marvinbryantia* (F(1,27) = 7.407; *p* = 0.011), *Sporosarcina* (F(1,27) = 10.170; *p* = 0.004), *Turibacter* (F(1,27) = 15.22; *p* = 0.001), *Enterococcus* (F(1,27) = 6.766; *p* = 0.015), *Enterorhabdus* (F(1,27) = 5.444; *p* = 0.027) and *Acetatifactor* (F(1,27) = 6.77; *p* = 0.015). Significant interaction effects were observed for *Lactococcus* (F(1,27) = 5.43; *p* = 0.028), *Limosilactobacillus* (F(1,27) = 12.34; *p* = 0.0016), *Enterococcus* (F(1,27) = 7.008; *p* = 0.013), *Marvinbryantia* (F(1,27) = 8.424; *p* = 0.007), *Romboutsia* (F(1,27) = 4.163; *p* = 0.051), *Sporosarcina* (F(1,27) = 13.940; *p* = 0.001) and *Turicibacter* (F(1,27) = 4.296; *p* = 0.048).

### 3.4. Dietary modulation of circulatory microbial-derived metabolism by Neurosyn240 is related to the gut microbiome

**Figure 4.**
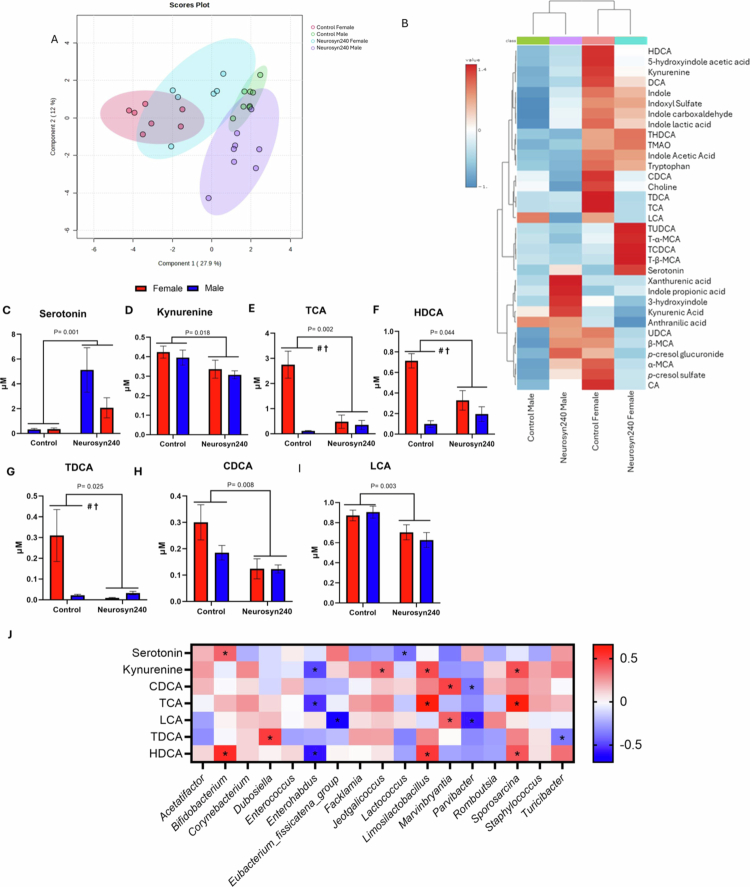
Neurosyn240 significantly modulated the circulatory metabolome profile which correlated with changes in the gut microbiome composition. (A) Partial least squares-discriminant analysis (PLS-DA) plot of the metabolomic profiles. (B) Heatmap displaying changes in concentrations of metabolites between the groups. (C−I) Concentrations of microbial-derived metabolites significantly modulated by the main effect of diet. *P*-value indicates the statistical significance of the main effect of diet. # denotes a significant main effect of sex. † indicates a significant interaction between sex and diet (*p* < 0.05). (J) Spearman rank correlation analysis between significantly modulated metabolites by diet and gut microbiome genera (FDR q < 0.05).

Targeted metabolomic profiling was conducted to gain insight into possible shifts in the production of bioactive metabolites associated with cognitive health. PLS-DA analysis showed a shift in metabolic response to the Neurosyn240 diet in both males and females (Figure 4A). This was further supported by heatmap analysis, which demonstrated alterations in the relative abundance of 33 metabolites in response to the Neurosyn240 diet compared to the control in males and females (Figure 4B). Seven metabolites were significantly impacted by the main effect of diet, including serotonin (F(1, 27) = 13.14; *p* = 0.001; Figure 4C), kynurenine (F(1,27) = 6.46; *p* = 0.018; Figure 4D), taurocholic acid (TCA; F(1, 24) = 11.44; *p* = 0.002; Figure 4E), hyodeoxycholic acid (HDCA; F(1, 27) = 4.51; *p* = 0.044; Figure 4F), taurodeoxycholic acid (TDCA; F(1, 27) = 5.65; *p* = 0.025; Figure 4G), chenodeoxycholic acid (CDCA; F(1, 27) = 8.41;p = 0.008; Figure 4H) and lithocholic acid (LCA; F(1, 27) = 11.11; *p* = 0.003; Figure 4I). See Supplementary Table S4 for all metabolite concentrations. To assess the relationship between dietary modulation of the gut microbiome and the circulatory metabolome, Spearman rank correlation analysis was performed on metabolites and microbiota genera modulated by the main effect diet (*p* < 0.05). This analysis revealed a significant correlation between the peripheral metabolome and the gut microbiome (Figure 4J). Procrustes analysis was conducted to evaluate the congruence of the two datasets. Significant similarities were observed between males (R = 0.69; *p* < 0.001; Supplementary Figure S2A) and females (R = 0.64; *p* < 0.001; Supplementary Figure S2B) on control and Neurosyn240 diet, suggesting similarity between microbiome and metabolome profiles.

A main effect of sex was observed on numerous tryptophan-related metabolites, including tryptophan (F(1,27) = 35.07; *p* < 0.001), 5-hydroxindole-acetic acid (5HIAA; F(1, 27) = 11.93; *p* = 0.002), anthranilic acid (F(1,27) = 10.43; *p* = 0.004), kynurenic acid (F(1,27) = 5.20; *p* = 0.031), indole acetic acid (IAA; F(1,27) = 89.41; *p* < 0.001), indole-3-lactic acid (ILA; F(1,27) = 10.12; *p* = 0.004), indole-3- carboxaldehyde (I3A; F(1,27) = 8.69; *p* = 0.007), indole (F(1,27) = 12.72; *p* = 0.002), indoxyl sulfate (F(1,27) = 10.28; *p* 0.004) (Supplementary Figure S3). Trimethylamine-*N*-oxide (TMAO; F(1,27) = 23.92; *p* < 0.001), THDCA; (F(1,24) = 7.14; *p* = 0.013), cholic acid (CA; F(1,27) = 7.01; *p* = 0.014), HDCA (F(1,27) = 30.39; *p* < 0.001), DCA; F(1,27) = 32.58; *p* < 0.001), TCA (F(1,27) = 21.24; *p* < 0.001) and TDCA (F1,27) = 4.74; *p* = 0.039) were also significantly influenced by sex (Supplementary Table S4). A significant interaction between sex and diet was observed in 5HIAA (F(1,27) = 5.52; *p* = 0.027), *p-*cresol sulfate (F(1,27) = 9.18; *p* = 0.006), *p-*cresol glucuronide (F(1,27) = 26.31; *p* < 0.001), alpha-muricholic acid (*α*-MCA; F(1,27) = 5.14; *p* = 0.032), CA (F(1,27) = 12.44; *p* = 0.002), ursodeoxycholic acid (UDCA; F(1,27) = 5.42; *p* = 0.028), HDCA (F(1,27) = 12.47; *p* = 0.002), DCA (F(1,27) = 6.47; *p* = 0.018), TCA (F(1,27) = 17.51; *p* = 0.003) and TDCA (F(1,27) = 6.52; *p* = 0.017) (Supplementary Table S4).

### 3.5. 5xFAD mice fed a Neurosyn240 diet showed reduced amyloid deposits and microglia in the hippocampus

**Figure 5.**
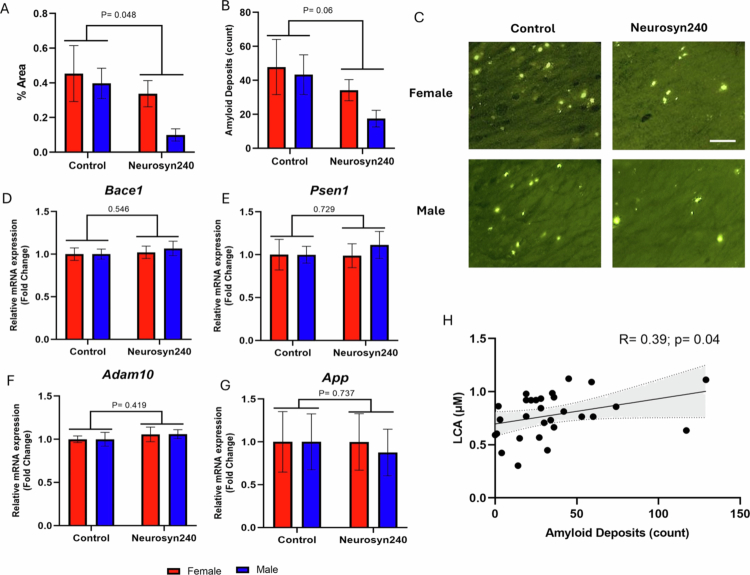
Mice consuming a Neurosyn240 diet have reduced amyloid deposits in the hippocampus. (A) The percentage of the hippocampal area filled with amyloid deposits showed a trend toward significance for the main effect of diet. (B) The main effect of diet significantly modulated the number of amyloid deposits within the hippocampus. Bars represent SEM. (C) Representative images of the hippocampus of control and Neurosyn240 mice at x10 magnification. Scale bar represents 100 µM. (D) Spearman rank correlation between circulatory LCA concentrations and the number of amyloid deposits in the hippocampus.

In the hippocampus, there was a significant effect of diet on the percentage area of amyloid deposits (F(1,27) = 4.33; *p* = 0.048; Figure 5A) and a near significant effect of diet on the number of amyloid deposits (F(1,27) = 3.68; *p* = 0.06; Figure 5B-C). However, there was no effect of sex on the percentage of amyloid deposits (F(1,27) = 2.20; *p* = 0.15) or number of amyloid deposits (F(1,27) = 1.06; *p* = 0.31). Similarly, no interaction was observed between sex and diet on the percentage area of amyloid deposits (F(1,27) = 0.83; *p* = 0.37) or number of amyloid deposits (F(1,27) = 0.35; *p* = 0.56). qPCR analysis showed no significant effects of diet on hippocampal expression of APP-processing genes, including presenilin 1 (*Psen1*; F(1,27) = 0.12; *p* = 0.73), beta-site APP-cleaving enzyme 1 (*Bace1*; F(1,27) = 0.37; *p* = 0.55), amyloid beta precursor protein (*App*; F(1,27) = 0.04; *p* = 0.85), and ADAM metallopeptidase domain 10 (*Adam10*; F(1,27) = 0.71; *p* = 0.41) (Figure 5D–G). There were no significant effects of sex or sex × diet interactions (*p* > 0.05).

Given the absence of a significant main effect of sex or interaction between sex and diet, correlation analysis was conducted on all mice as a single group to examine the relationship between metabolites significantly modulated by diet (*p* < 0.05) and amyloid deposits (Supplementary Figure S4). A significant positive association was found between serum LCA concentrations and the number of amyloid deposits in mice (r = 0.39; *p* = 0.04), suggesting that these changes may be linked to metabolite levels (Figure 5H).

**Figure 6.**
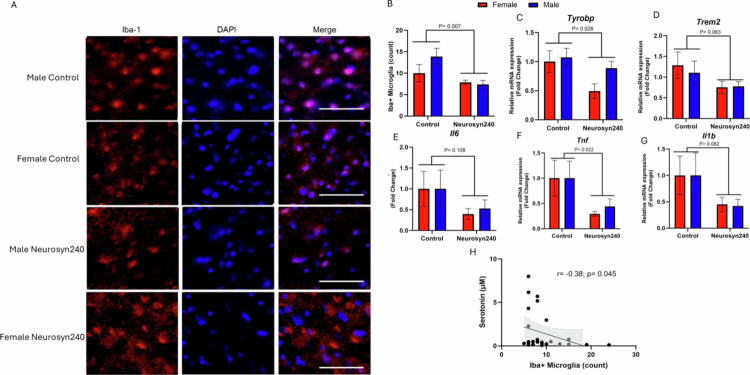
Mice consuming a Neurosyn240 diet have reduced Iba-1 positive microglia in the hippocampus. (A) Representative images of Iba-1 positive microglia in the hippocampus of control and Neurosyn240 mice at x10 magnification. Scale bar represents 100 µM. (B) Iba-1 positive microglia within a random 200 µm^2^ region of interest (ROI) within the hippocampus by sex and treatment groups. Relative fold change in mRNA gene expression of *Tyrobp* (D)*, Trem2* (B)*, Il6* (E), *Tnf* (F) and *Il1b* (G) in Neuroyn240 compared to sex-matched control groups. *P*-value represents the statistical significance of the main effect of diet. Error bars represent SEM. (H) Spearman rank correlation between circulatory serotonin concentrations and Iba-1 positive microglia in 5xFAD mice.

In the hippocampus, a significant main effect of diet was observed on the number of Iba-1 positive microglia (F(1,27) = 8.55; *p* = 0.007; Figure 6A-B), but no effect of sex (F(1,27) = 1.31; *p* = 0.26) or interaction between sex and diet (F(1,27) = 2.20; *p* = 0.15). Consistent with this, qPCR analysis revealed a significant effect of diet on the expression of markers of disease-associated microglia (DAM), triggering receptor expressed on myeloid cells 2 (*Trem2;* F(1,27) = 5.49; *p* = 0.03) and a near significant effect on TYRO protein tyrosine kinase binding protein (*Tyrobp*; F(1,27) = 3.78; *p* = 0.06) (Figure 6C-D). Furthermore, there was a significant effect of diet on the proinflammatory cytokine tumor necrosis factor (*Tnf*) expression (F(1,27) = 5.96; *p* = 0.02), a near-significant effect on interleukin 1 beta (*Il1b*; F(1,27) = 3.79; *p* = 0.062), and no significant effect on interleukin 6 (*Il6*; F(1,27) = 2.77; *p* = 0.11) (Figure 6E–G). There were no significant effects of sex or sex × diet interactions (*p* > 0.05). The number of hippocampal Iba-1 positive microglia negatively correlated with circulatory serotonin concentrations (r = −0.38; *p* = 0.045), suggesting a possible gut-brain interaction (Figure 6H).

In addition to microglial markers, hippocampal expression of genes linked to neuronal health and AD pathology was assessed. VGF Nerve Growth Factor Inducible (*Vgf*) and the dual-specificity phosphatases Dual Specificity Phosphatase 4 (*Dusp4*) and Dual Specificity Phosphatase 6 (*Dusp6*), which have been implicated in amyloid clearance and memory function, were measured by qPCR.^51-53^ There was no effect of diet, sex or interaction between sex and diet for *DUSP4* (F(1,27) = 0.76; *p* = 0.39), *DUSP6* (F(1,27) = 0.55; *p* = 0.47) or *Vgf* (F(1,27) = 0.55; *p* = 0.47) (Supplementary Figure S5).

### 3.6. Neurosyn240 differently affects hippocampal gene expression

**Figure 7.**
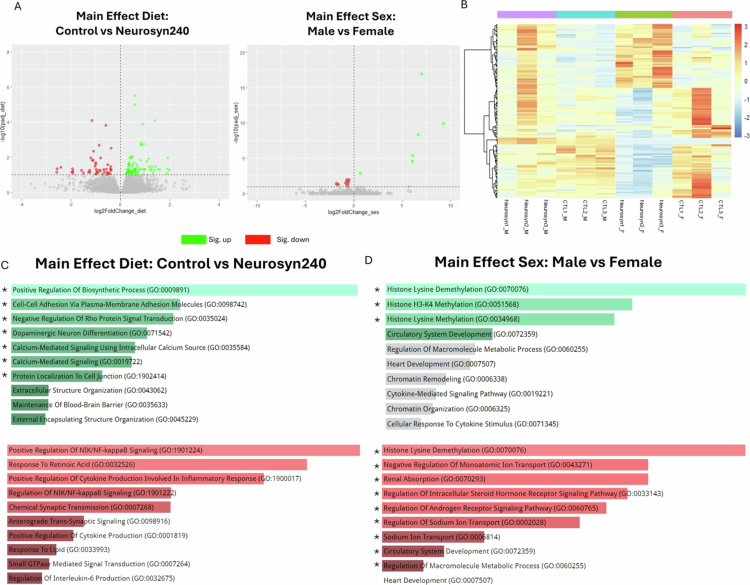
Neurosyn240 significantly modulated gene expression within the hippocampus. (A) Volcano plot showing differentially expressed genes from the main effect of diet (left) or sex (right). (B) Heatmap displaying the expression of the differentially expressed genes between treatment groups. (C) Gene Ontology pathway analysis of genes significantly upregulated (green) and downregulated (red) by the main effect of diet. (D) Gene Ontology analysis of genes significantly upregulated (green) and downregulated (red) by the main effect of sex. Red or green pathway color indicates *p* < 0.05. Gray pathway indicates *p* > 0.05. * = FDR adjusted q < 0.05.

To examine the potential neuroprotective mechanisms of Neurosyn240, RNA sequencing analysis was conducted on the hippocampal brain region, a region significantly affected by AD.^54^ Using DESeq2 analysis, we identified 47 DEGs that were upregulated and 77 genes that were downregulated by diet (Figure 7A). Additionally, 7 genes were upregulated, and 7 genes were downregulated by sex. Hierarchical clustering analysis displayed the expression of significant DEGs (Figure 7B). GO analysis highlighted the functional properties of DEGs. Neurosyn240 upregulated processes related to neuronal health, including positive regulation of biosynthetic processes (GO:0009891) and dopaminergic neuron differentiation (GO:0071542), critical for maintaining neuronal function (Figure 7C).^55^^,^^56^ Notably, pathways related to calcium-mediated signaling (GO:0019722, GO:0035584) and protein localization to cell junctions (GO:1902414) were significantly upregulated, suggesting synaptic signaling and cellular communication may be improved (P_FDR_ < 0.1). Several key inflammatory and immune-related pathways were downregulated by Neurosyn240 consumption (*p* < 0.05). Additionally, NF-kappaB signaling (GO:1901224), a central regulator of inflammation, along with pathways involved in the positive regulation of cytokine production (GO:1900017), were downregulated. However, it should be noted that these pathways did not reach significance at an adjusted *p*-value < 0.1.

GO analysis revealed sexually dimorphic effects on histone modifications and signaling pathways (Figure 7D). Females showed upregulation of H3-K4 and histone lysine methylation, downregulation of histone lysine demethylation, and reduced androgen and steroid hormone receptor signaling compared to males, aligning with previous studies.^57^^,^^58^

To investigate potential pathways underlying hippocampal reductions in amyloid deposits, gene expression results were compared to a curated set of 155 genes associated with amyloid beta formation, binding, and clearance, identifying those consistently present across two functional genome databases.^59-61^ DESeq2 GLM analysis showed that the Neurosyn240 significantly (P_FDR_ < 0.1) upregulated expression of genes associated with amyloid beta binding (Clusterin (*Clu*)*;* P_FDR_ = 0.051), clearance (Low-Density Lipoprotein Receptor-Related Protein 2 (*Lrp2*)*;* P_FDR_ = 0.045) and cellular response (Vascular Cell Adhesion Molecule 1 (*Vcam1*)*;* P_FDR_ = 0.027), while downregulating the amyloid binding gene *Ager* P_FDR_ = 0.07 (Table 1; Supplementary Table S5 for full list of gene expression). No genes associated with amyloid beta formation, binding or clearance were significantly modulated by sex (Supplementary Table S6). Correlating genes and circulatory metabolites significantly modulated by diet highlighted a negative correlation between *Lrp2* and LCA concentrations (r = −0.60; *p* = 0.041; Supplementary Figure S6), suggesting a potential link between the metabolome and the brain.

**Table 1.** Amyloid-associated gene expression modulated by the main effect of diet (Control vs Neurosyn240).

EntrezID	Gene	Description	Category	Log_2_FC	Adjusted *p*-value
22329	*Vcam1*	von Hippel-Lindau binding protein 1	cellular response to amyloid-beta	0.51	0.027
14725	*Lrp2*	low-density lipoprotein receptor-related protein 1	amyloid-beta clearance	1.73	0.045
12759	*Clu*	clusterin	amyloid-beta binding	0.85	0.051
11596	*Ager*	advanced glycosylation end product-specific receptor	amyloid-beta binding	−1.32	0.077

## 4. Discussion

The bioactive constituents of a Mediterranean-style diet are well-recognized for their neuroprotective properties.^62^ However, the mechanisms through which they may slow or reduce AD pathology are currently unclear. Using the 5xFAD mouse model of AD, we evaluated the protective effects of a Mediterranean diet-inspired bioactive blend, Neurosyn240, on modulating metabolite communication within the microbiota-gut-brain axis in both sexes during the prodromal stage of decline.^63-65^ Our results highlight novel protective effects of Neurosyn240 in mitigating early AD-related neuropathology in 5xFAD mice. Neurosyn240 consumption over twelve weeks significantly shifted the gut microbiome profile, which was associated with the modulation of the circulatory microbial-derived metabolome, including increased serotonin and reduced LCA levels. These changes were associated with reduced hippocampal Iba-1 positive microglia and amyloid deposits.

The 5xFAD model typically exhibits hippocampal-dependent cognitive deficits from 6 months of age,^20^ with some studies reporting only mild deficits as late as 13 months.^21^ At 5 months, a prodromal stage of AD in this model,^20^ no significant difference in cognitive behavior was present between the control and Neurosyn240 diet groups. However, both males and females showed trends towards reduced anxiety-related behavior in the open field assessment following Neurosyn240 consumption. Given that anxiety is an early sign of AD, preceding cognitive decline,^66^ these findings may suggest effects on early disease manifestations. Previous reports on C57BL/6J wild-type mice exposed to chronic low-grade inflammation suggested that Neurosyn240 consumption improved recognition memory, supporting potential cognitive benefits.^29^ In parallel with these behavioral trends, amyloid deposits in the hippocampus were also reduced with Neurosyn240 intake, indicating that while behavioral differences are subtle at this stage, there are early effects on amyloid pathology. This suggests that the diet may influence early disease processes before pronounced cognitive deficits emerge. Consistent with previous research in 5xFAD mice,^67^ no significant sex differences were detected across behavioral assessments. These outcomes are consistent with human imaging studies in which Mediterranean diet adherence correlated with lower amyloid burden even before cognitive symptoms become apparent.^68^^,^^69^

Metabolite-mediated signaling within the microbiota-gut-brain axis is increasingly recognized as an important contributor to the neuroprotective effects associated with the bioactive constituents of the Mediterranean diet.^18^ Neurosyn240 consumption did not result in significant alterations in microbial alpha diversity but was associated with significant changes in beta diversity, indicating shifts in overall community composition. Neurosyn240 may therefore not induce widespread changes in overall microbiota diversity but instead modulate specific microbial taxa and associated metabolic functions. Neurosyn240 significantly reduced concentrations of TCA, HDCA and LCA, bile acids which are typically considered cytotoxic and are elevated in AD patients,^70^ and clinical trials have similarly reported that adherence to Mediterranean-style diets can lower circulating secondary bile acids such as LCA and DCA.^71^ The bile salt hydrolase (BSH)-capable *Limosilactobacillus,*^72^^,^^73^ which can deconjugate bile acids, showed a positive correlation with reductions in TCA and HDCA. *Dubosiella,* a bacterium with 7-*α*-dehydroxylation capability to convert primary bile acids into secondary bile acids,^74^ was also significantly decreased with Neurosyn240 consumption and correlated with reductions in TDCA. The correlation between these microbial changes and metabolite levels suggests gut microbiome modulation may play a critical role in the observed metabolic shifts. Increases in the beneficial microbe, *Parvibacter,* by Neurosyn240 inversely correlated with LCA concentrations. *Parvibacter* abundance, which can be increased by vitamin intake,^75^ upregulates the *baiA* gene, enhancing the metabolism of LCA to the less toxic form, 3-oxo-5beta-cholanate.^76^ Given that LCA can accumulate in the brain of AD patients due to increased serum levels and is associated with cognitive decline,^70^ these reductions induced by Neurosyn240 may offer neuroprotective benefits.

Neurosyn240 significantly reduced amyloid deposits in the hippocampus of 5xFAD mice, aligning with previous longitudinal human studies.^77^ Reductions in LCA were associated with decreased amyloid deposits in the hippocampus, suggesting a possible connection between the peripheral metabolome and the brain. Hippocampal RNA sequencing revealed upregulation of Aβ clearance-related genes, including *Lrp2* and *Clu*, in mice receiving Neurosyn240. LRP2, a key Aβ transporter at the BBB, plays a critical role in clearing Aβ from the brain through binding with CLU.^78^ In non-neuronal tissues, LCA can strongly inhibit LRP2 expression *in vitro*^79^ via the activation of the vitamin D receptor (VDR),^80^ suggesting that the observed reductions in LCA may enhance the expression of LRP2, facilitating the clearance of Aβ from the brain. However, this mechanism has not previously been demonstrated in the brain, and these findings propose this association within the microbiota-gut-brain axis context for the first time. However, these findings align with evidence linking Mediterranean diet adherence to reduced Aβ accumulation via interactions with genes such as *Clu.*^77,81^

Neurosyn240 increased peripheral serotonin levels in comparison to controls. With approximately 95% of serotonin located in the periphery through tryptophan metabolism, its dysregulation is considered an early sign of AD.^42^ AD patients often exhibit reduced serum serotonin levels^82^ and dysregulated tryptophan metabolism,^83^ which can exacerbate inflammation.^84-86^ Elevated circulatory serotonin following Neurosyn240 intake may reduce these effects. Notably, increased serotonin levels may be linked to the abundance of *Bifidobacterium*, which positively correlated with serotonin concentrations. Strains of *Bifidobacterium,* such as *Bifidobacterium dentium,* can stimulate serotonin release from enterochromaffin cells via secretion of short-chain fatty acids, including acetate.^87^ In addition, *Bifidobacterium* can influence serotonin biosynthesis by modulating tryptophan availability and metabolism.^88^ Conversely, serotonin itself can shape microbial community composition by regulating intestinal signaling and motility, suggesting a bidirectional relationship.^89^

Neurosyn240 intake significantly reduced the number of hippocampal Iba-1 positive microglia. Phenolic components of the Mediterranean diet have previously been associated with mitigating microglial responses in the brain.^90^ However, the specific mechanisms are poorly understood. Reductions in amyloid deposits in the hippocampus may decrease microglial activation, as these cells often respond to amyloid accumulation and can promote inflammation. Consistent with these findings, hippocampal expression of the microglia-associated proinflammatory cytokine gene *Tnf* was significantly reduced by Neurosyn240, while *Il1b* showed a near-significant decrease. The reduction in Iba-1 positive microglia may decrease proinflammatory signaling in the hippocampus.^91^ Adherence to the Mediterranean diet has been linked to lower levels of inflammatory cytokines, including IL-6 and TNF-*α*, suggesting its potential role in modulating neuroinflammation.^92^^,^^93^ In line with this, hippocampal expression of markers of disease-associated microglia (DAM) was reduced, indicating a diminished DAM response. Deficiency or loss-of-function of TREM2 and its adapter TYROBP/DAP12 has been shown to reduce plaque-associated microglial density and impair DAM formation.^94^^,^^95^ This reduction may reflect the lower amyloid plaque burden observed, contributing to reduced pro-inflammatory signaling.

Peripheral serotonin concentrations inversely correlated with the number of Iba-1 positive microglia. Although peripheral serotonin cannot cross the blood-brain barrier, it can modulate central neuroinflammation via vagus nerve signaling, activating brainstem nuclei and influencing serotonergic and noradrenergic neurons.^96^ Vagus nerve activation can suppress pro-inflammatory cytokines (TNF-*α*, IL-1β, IL-6), in line with our observed reductions in hippocampal cytokine expression, and promote anti-inflammatory signaling, including a shift of microglia from a pro-inflammatory M1 to an anti-inflammatory M2 phenotype.^97^ Serotonin is also a potent immune modulator through receptors expressed on immune cells, inhibiting pro-inflammatory cytokine production, including TNF-*α* and IL-1 *β*.^98^ Microglial cells, as resident macrophage-like cells of the central nervous system, respond to signals from the peripheral immune system and induce neuroinflammation through the activation of pro-inflammatory cytokines.^99^ In AD, neuroinflammation plays a critical role in pathogenesis by increasing microglial cell and astrocyte activation. Dysregulation of the serotonergic system in AD patients can influence neuroinflammation, and immune system and microglial activation.^100^ As such, Neurosyn240-induced modulation of *Bifidobacterium*, serotonin, and Iba-1 microglia may play a neuroprotective role. These findings support human data suggesting that Mediterranean-style diets may influence systemic inflammation and indirectly modulate neuroinflammatory processes.^101^

Although certain circulatory metabolites and microbial genera exhibited sex differences, generally both sexes responded similarly to Neurosyn240 consumption, particularly in the brain. No significant sex differences were observed in the modulation of AD-related neuropathologies. Conflicting evidence exists regarding sex differences in response to Mediterranean diet interventions, with some studies reporting significant sex-specific effects,^102,103^ while others find no such difference.^104-107^ Previous reports suggest neuropathological outcomes may not be as strongly influenced by sex as those related to functional connectivity.^104^ Autopsy studies also demonstrate that amyloid beta plaque load in the hippocampus is similar in men and women,^108^ implying that sex may have a more limited impact on the accumulation of key pathological markers, such as amyloid pathology.

We recognize that our study has limitations, and further work will be required. Despite targeting some of the top metabolites currently associated with neuronal health, the gut microbiome is capable of directly and indirectly modifying a variety of metabolites, which may contribute to the protective effects of Neurosyn240. For example, short-chain fatty acids produced from the microbiota through the anaerobic fermentation of indigestible polysaccharides are protective against AD.^109^ The 5xFAD mouse model is widely utilized in AD research due to replicating key pathological features observed in human AD.^67,110^ As such, this model uniquely enables the assessment of early dietary interventions within a prodromal disease progression at a relatively young age (5 months). While the model effectively recapitulates amyloid pathology, it does not capture the tau-related neurofibrillary tangles characteristic of later stages of AD. Therefore, this study could not assess the modulation of tau phosphorylation; an aspect that has previously been found to be improved by anthocyanins, a (poly)phenol present in the Neurosyn240 diet.^111^ As such, further *in vivo* work with appropriate tau transgenic models may be necessary. Furthermore, our findings are based on an integrated multi-omics approach combining metabolomics, RNA sequencing, and qPCR; therefore, further work will be required to establish the causality of the underlying mechanisms. RNA sequencing analysis was also limited to three biological replicates per group and sex, which may limit statistical power. Finally, future studies may also look to extend Neurosyn240 supplementation to older 5xFAD mice with established AD neuropathology to determine whether its protective effects translate to later stages of disease progression and cognitive outcomes.

## 5. Conclusions

Overall, our results indicate novel interactions between a Mediterranean diet-inspired supplement and a neuroprotective response against early AD-associated pathology. This protective effect appears to be associated with metabolite-driven pathways of the microbiota-gut-brain axis. These findings enhance our understanding of how dietary components can influence brain health and support the potential benefits of therapeutic interventions during the prodromal stage of AD.

## Supplementary Material

Supplementary material Supplementary_final rev2.

## Data Availability

The 16S rRNA gene sequence data have been deposited in the NCBI BioProject database (https://www.ncbi.nlm.nih.gov/bioproject/) under accession number PRJNA1169679. Raw RNA sequences have been deposited NCBI BioProject database under accession number PRJNA1320695. Other original data will be made available from the corresponding author on reasonable request.

## Abbreviations

Aβ: Amyloid Beta
AD: Alzheimer's Disease
Ager: Advanced Glycation End-products Receptor Gene
APP: Amyloid Precursor Protein
BBB: Blood-Brain Barrier
BW: Body Weight
CA: Cholic Acid
CDCA: Chenodeoxycholic Acid
CLU: Clusterin
DCA: Deoxycholic Acid
DEGs: Differentially Expressed Genes
FDR: False Discovery Rate
GLM: Generalized Linear Model
GO: Gene Ontology
HDCA: Hyodeoxycholic Acid
I3A: Indole-3-Carboxaldehyde
IAA: Indole Acetic Acid
ILA: Indole-3-Lactic Acid
IL-1 β: Interleukin-1 β
LCA: Lithocholic Acid
LC-MS/MS: Liquid chromatography with tandem mass spectrometry
LDA: Linear Discriminant Analysis
LEfSe: Linear Discriminant Analysis Effect Size
Lrp2: Low-Density Lipoprotein Receptor-Related Protein 2
MCA: Muricholic Acid
NF-kappaB: Nuclear Factor Kappa-Light-Chain-Enhancer of Activated B Cells
NOR: Novel Object Recognition
OF: Open Field
OUT: Operational Taxonomic Units
PCoA: Principal Coordinates of Analysis
PERMANOVA: Permutational Multivariate Analysis of Variance
PLS-DA: Partial Least Squares Discriminant Analysis
TCA: Taurocholic Acid
TDCA: Taurodeoxycholic Acid
TMAO: Trimethylamine-*N*-oxide
TNF-α: Tumor Necrosis Factor-α
UDCA: Ursodeoxycholic Acid
VCAM1: Vascular Cell Adhesion Molecule 1
VDR: Vitamin D Receptor
