# Supplementary material for: A novel Mediterranean diet-inspired supplement reduces hippocampal amyloid deposits and microglial activation through the modulation of the microbiota gut-brain axis in 5xFAD mice
Source: Gut Microbes. 2026 Jan 13;18(1):2614030. doi: 10.1080/19490976.2026.2614030 (PMC12802989; doi:10.1080/19490976.2026.2614030)
Supplement: Supplementary material — Supplementary_final rev2. [file KGMI_A_2614030_SM2740.docx]

| **GenBank Accession** | **Gene** | **Description** | **Forward primer sequence**  **(5’ -> 3’)** | **Reverse primer sequence**  **(5’ -> 3’)** |
| --- | --- | --- | --- | --- |
| NM_007563 | ***Bace1*** | Beta-site APP-cleaving enzyme 1 | TGCTGCCATCACTGAATCGGAC | GGAATGTGGGTCTGCTTCACCA |
| NM_007419 | ***Psen1*** | Presenilin 1 | GAGACTGGAACACAACCATAGCC | AGAACACGAGCCCGAAGGTGAT |
| NM_007419 | ***Adam10*** | ADAM metallopeptidase domain 10 | TGCACCTGTGCCAGCTCTGATG | GATAGTCCGACCACTGAACTGC |
| NM_007471 | ***App*** | Amyloid beta precursor protein | TCCGTGTGATCTACGAGCGCAT | GCCAAGACATCGTCGGAGTAGT |
| NM_031168 | ***Il6*** | Interleukin 6 | TACCACTTCACAAGTCGGAGGC | CTGCAAGTGCATCATCGTTGTTC |
| NM_008361 | ***Il-1b*** | Interleukin 1 beta | TGGACCTTCCAGGATGAGGACA | GTTCATCTCGGAGCCTGTAGTG |
| NM_013693 | ***Tnf*** | Tumor necrosis factor | AAATGGGCTCCCTCTCATCAGTTC | TCTGCTTGGTGGTTTGCTACGAC |
| NM_026268 | ***Dusp4*** | Dual specificity phosphatase 4 | CTCCTGGTTCATGGAAGCCATC | GACGAACTCAAAAGCCTCCTCC |
| NM_026269 | ***Dusp6*** | Dual specificity phosphatase 6 | CTCGGATCACTGGAGCCAAAAC | TCTGCATGAGGTACGCCACTGT |
| NM_001039385 | ***Vgf*** | VGF nerve growth factor inducible | CTTTGACACCCTTATCCAAGGCG | GCTAATCCTTGCTGAAGCAGGC |
| NM_011662 | ***Tyrobp*** | TYRO protein tyrosine kinase binding protein | GTGACTTGGTGTTGACTCTGCTG | GATAAGGCGACTCAGTCTCAGC |
| NM_031254 | ***Trem2*** | Triggering receptor expressed on myeloid cells 2 | CTACCAGTGTCAGAGTCTCCGA | CCTCGAAACTCGATGACTCCTC |
| *NM_013556* | ***Hprt1*** | Hypoxanthine phosphoribosyltransferase 1 | CTGGTGAAAAGGACCTCTCGAAG | CCAGTTTCACTAATGACACAAACG |
| *NM_013684* | ***Tbp*** | TATA-box binding protein | CTACCGTGAATCTTGGCTGTAAAC | AATCAACGCAGTTGTCCGTGGC |

**Supplementary Table S1: Primer sequences**

**Supplementary Table S2: Beta diversity pairwise PERMANOVA analysis.** FDR= Benjamini-Hochberg procedure

| Pair | F-Value | R-Squared | P-value | FDR |
| --- | --- | --- | --- | --- |
| Control_Female vs Control_Male | 1.948 | 0.130 | 0.083 | 0.093 |
| Control_Female vs Neurosyn240_Male | 5.999 | 0.316 | 0.007 | 0.021 |
| Control_Female vs Neurosyn240_Female | 1.978 | 0.132 | 0.093 | 0.093 |
| Control_Male vs Neurosyn240_Male | 4.391 | 0.239 | 0.003 | 0.018 |
| Control_Male vs Neurosyn240_Female | 2.108 | 0.131 | 0.059 | 0.0885 |
| Neurosyn240_Male vs Neurosyn240_Female | 3.300 | 0.191 | 0.024 | 0.048 |

**
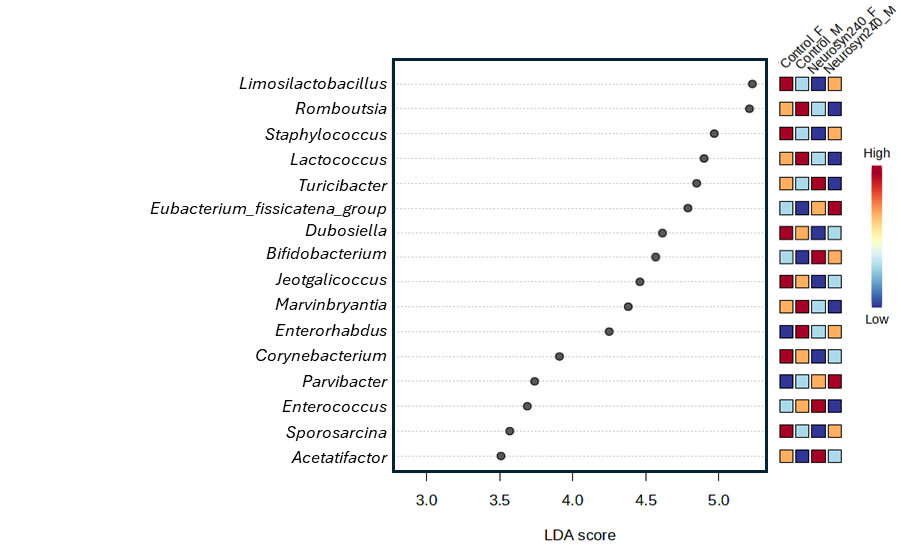
**

**Supplementary Figure S1: Linear discrimination analysis (LDA) effect size (LEfSe) revealed significant differences in 16 genera between groups at the genus level.**

**Supplementary Table S3: Microbiome abundance counts. P-values generated by two-way ANOVA between sex (males and females) and diet (control and Neurosyn240) and interaction. Bold values= p<0.05.**

| Metabolite | Control Female | | Control Male | | Neurosyn240 Female | | Neurosyn240 Male | | Source of Variation | | | | | |
| --- | --- | --- | --- | --- | --- | --- | --- | --- | --- | --- | --- | --- | --- | --- |
|  |  |  |  |  |  |  |  |  | **Diet** | | **Sex** | | **Interaction** | |
|  | **Mean** | **SD** | **Mean** | **SD** | **Mean** | **SD** | **Mean** | **SD** | **F** | **p** | **F** | **p** | **F** | **p** |
| *Lactococcus* | 12263.71 | 7723.70 | 5811.00 | 4919.62 | 9370.50 | 11298.46 | 3017.83 | 3571.01 | 6.666 | **0.016** | 4.287 | **0.048** | 5.434 | **0.028** |
| *Limosilactobacillus* | 2453.29 | 2067.80 | 0.38 | 0.74 | 0.00 | 0.00 | 4.00 | 8.00 | 12.12 | **0.0017** | 12.11 | **0.0017** | 12.34 | **0.0016** |
| *Marvinbryantia* | 131.86 | 78.68 | 411.88 | 255.27 | 69.75 | 41.62 | 49.83 | 41.73 | 17.220 | **<0.001** | 7.407 | **0.011** | 8.424 | **0.007** |
| *Parvibacter* | 0.43 | 0.79 | 2.63 | 2.13 | 46.13 | 32.01 | 65.83 | 48.63 | 25.520 | **<0.001** | 1.778 | 0.194 | 1.312 | 0.262 |
| *Romboutsia* | 1476.43 | 1644.51 | 2591.63 | 1467.04 | 844.75 | 737.33 | 237.33 | 334.59 | 12.690 | **0.001** | 0.419 | 0.523 | 4.163 | 0.051 |
| *Dubosiella* | 3724.43 | 3705.44 | 2305.29 | 2088.94 | 11.50 | 13.53 | 369.00 | 352.86 | 13.140 | **0.001** | 0.832 | 0.370 | 1.947 | 0.174 |
| *Sporosarcina* | 56.43 | 46.81 | 0.88 | 1.81 | 0.00 | 0.00 | 3.17 | 5.00 | 10.880 | **0.003** | 10.170 | **0.004** | 13.940 | **0.001** |
| *Turicibacter* | 303.29 | 445.91 | 0.00 | 0.00 | 990.75 | 805.64 | 0.00 | 0.00 | 4.296 | **0.048** | 15.220 | **0.001** | 4.296 | **0.048** |
| *Acetatifactor* | 26.43 | 60.01 | 0.00 | 0.00 | 43.13 | 43.21 | 0.00 | 0.00 | 0.535 | 0.471 | 6.756 | **0.015** | 0.311 | 0.582 |
| *Corynebacterium* | 106.57 | 168.56 | 15.13 | 25.67 | 0.50 | 1.41 | 11.83 | 21.09 | 3.282 | 0.081 | 1.720 | 0.201 | 3.298 | 0.081 |
| *Enterococcus* | 29.86 | 27.58 | 30.50 | 28.06 | 76.00 | 66.04 | 2.33 | 1.51 | 0.434 | 0.516 | 6.766 | **0.015** | 7.008 | **0.013** |
| *Enterorhabdus* | 105.57 | 87.36 | 356.75 | 307.40 | 139.25 | 56.26 | 181.50 | 106.44 | 1.413 | 0.245 | 5.444 | **0.027** | 3.007 | 0.094 |
| *Eubacterium_fissicatena_group* | 180.29 | 333.22 | 0.25 | 0.71 | 578.88 | 351.07 | 773.83 | 1241.61 | 8.772 | **0.006** | 0.071 | 0.791 | 1.222 | 0.279 |
| *Facklamia* | 11.71 | 12.20 | 4.25 | 6.14 | 0.00 | 0.00 | 3.50 | 5.86 | 5.713 | 0.087 | 0.504 | 0.484 | 5.026 | 0.069 |
| *Jeotgalicoccus* | 412.57 | 738.38 | 47.50 | 85.51 | 0.63 | 1.77 | 28.00 | 34.98 | 2.753 | 0.109 | 1.660 | 0.209 | 2.560 | 0.121 |
| *Staphylococcus* | 1360.14 | 2229.76 | 139.00 | 244.89 | 15.50 | 27.23 | 143.00 | 169.76 | 2.382 | 0.134 | 1.500 | 0.231 | 3.709 | 0.065 |
| *Bifidobacterium* | 2004.43 | 2175.03 | 1863.13 | 2866.25 | 4536.25 | 3115.18 | 3131.17 | 3647.64 | 4.255 | **0.049** | 0.636 | 0.432 | 2.521 | 0.124 |


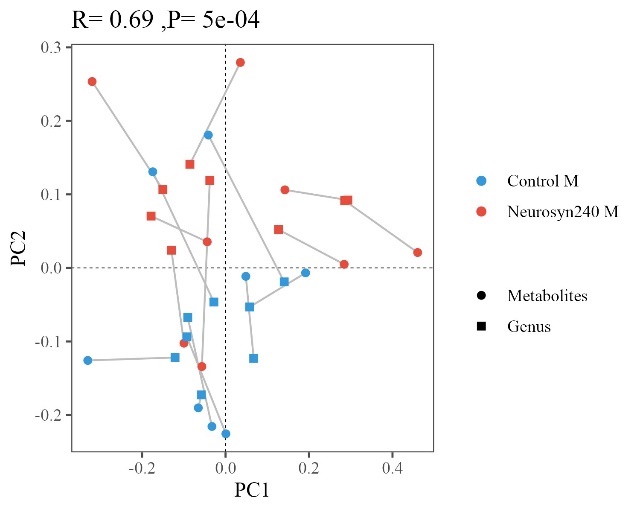


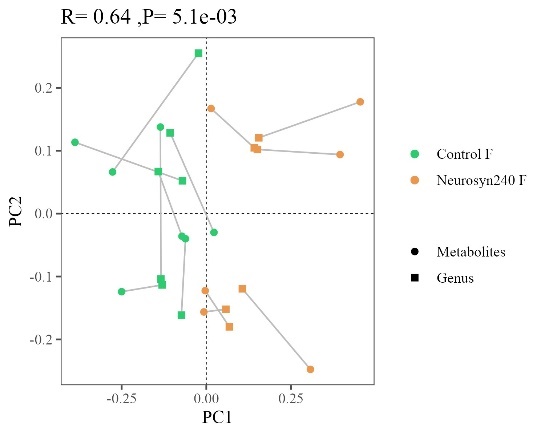


**Supplementary Figure S2: Procrustes analysis of the congruence of metabolite and microbiome profiles.**


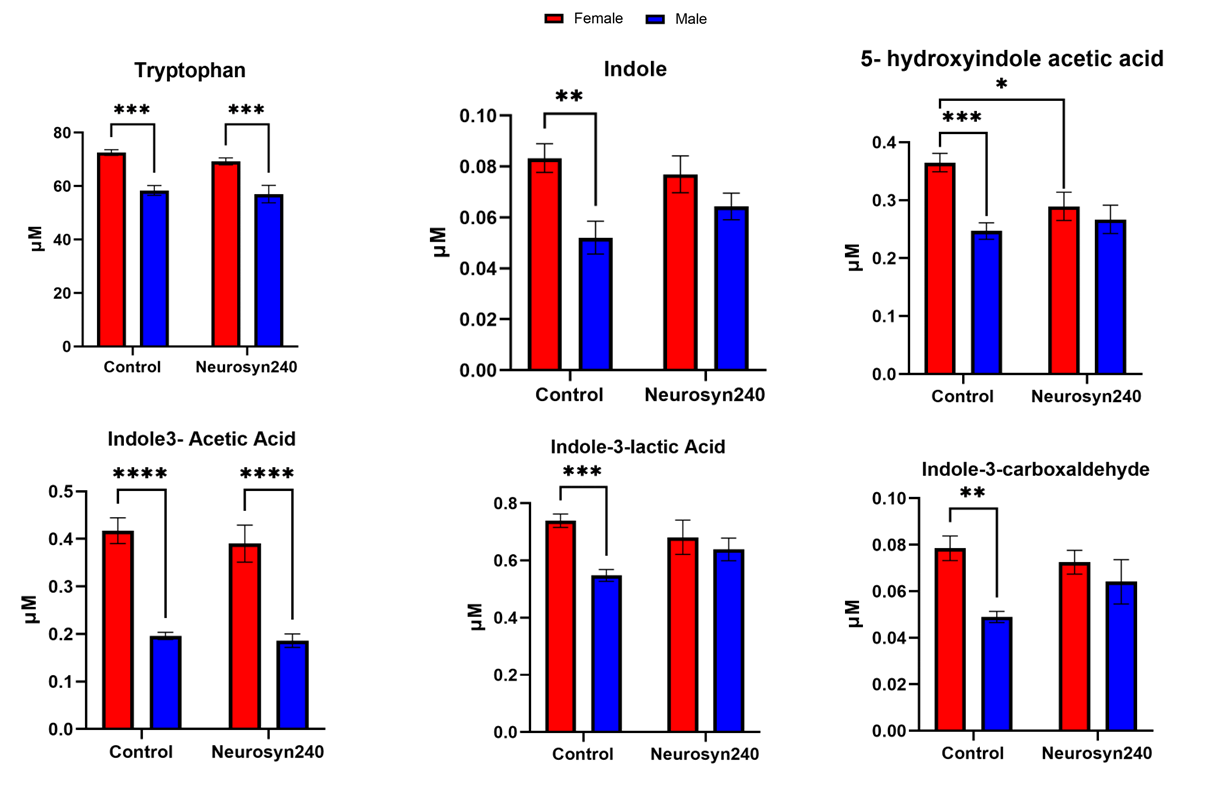


**Supplementary Figure S3: Tryptophan and indole metabolites significantly modulated by sex.**

| **Metabolite**  **Supplementary Table S4: Serum metabolite concentrations.** P-values generated by two-way ANOVA between sex (males and females) and diet (control and Neurosyn240) and interaction. Bold values= p<0.05. All concentrations are given in µM. | **Control Female**  **(µM)** | | **Control Male**  **(µM)** | | **Neurosyn240 Female**  **(µM)** | | **Neurosyn240 Male**  **(µM)** | | **Source of Variation** | | | | | |
| --- | --- | --- | --- | --- | --- | --- | --- | --- | --- | --- | --- | --- | --- | --- |
|  |  |  |  |  |  |  |  |  | **Diet** | | **Sex** | | **Interaction** | |
|  | **Mean** | **SD** | **Mean** | **SD** | **Mean** | **SD** | **Mean** | **SD** | **F** | **p** | **F** | **p** | **F** | **p** |
| Serotonin | 0.37 | 0.18 | 0.37 | 0.30 | 3.99 | 4.65 | 2.07 | 2.29 | 13.140 | **0.001** | 2.860 | 0.104 | 2.927 | 0.100 |
| Kynurenine | 0.42 | 0.08 | 0.28 | 0.04 | 0.34 | 0.11 | 0.31 | 0.06 | 6.459 | **0.018** | 0.687 | 0.415 | 0.001 | 0.993 |
| Tryptophan | 72.59 | 2.83 | 58.65 | 5.59 | 69.25 | 3.16 | 57.02 | 9.27 | 1.111 | 0.302 | 35.070 | **<0.001** | 0.194 | 0.663 |
| 5-Hydroxyindole Acetic Acid | 0.36 | 0.04 | 0.25 | 0.04 | 0.29 | 0.06 | 0.27 | 0.07 | 1.855 | 0.185 | 11.930 | **0.002** | 5.524 | **0.027** |
| Anthranilic Acid | 0.03 | 0.01 | 0.04 | 0.01 | 0.03 | 0.01 | 0.04 | 0.01 | 0.890 | 0.355 | 10.430 | **0.004** | 0.593 | 0.448 |
| Kynurenic Acid | 0.05 | 0.02 | 0.06 | 0.01 | 0.04 | 0.02 | 0.07 | 0.04 | 0.184 | 0.672 | 5.199 | **0.031** | 1.839 | 0.187 |
| p-Cresol Sulfate | 7.91 | 2.45 | 3.26 | 1.38 | 4.78 | 3.52 | 5.96 | 2.30 | 0.146 | 0.706 | 3.065 | 0.092 | 9.178 | **0.006** |
| p-Cresol Glucuronide | 1.37 | 0.33 | 0.55 | 0.21 | 0.71 | 0.74 | 1.66 | 0.40 | 1.390 | 0.249 | 0.299 | 0.589 | 26.310 | **<0.001** |
| Indole Acetic Acid | 0.42 | 0.07 | 0.19 | 0.02 | 0.39 | 0.10 | 0.19 | 0.04 | 0.681 | 0.417 | 89.410 | **<0.001** | 0.146 | 0.705 |
| Indole-3-Propionic Acid | 0.49 | 0.24 | 0.61 | 0.21 | 0.62 | 0.49 | 1.09 | 1.01 | 1.952 | 0.175 | 1.689 | 0.206 | 0.647 | 0.429 |
| Xanthurenic Acid | 0.03 | 0.02 | 0.03 | 0.01 | 0.03 | 0.02 | 0.07 | 0.08 | 1.483 | 0.235 | 1.202 | 0.283 | 1.546 | 0.225 |
| Indole-3-Lactic Acid | 0.74 | 0.06 | 0.56 | 0.06 | 0.68 | 0.15 | 0.64 | 0.11 | 0.201 | 0.658 | 10.120 | **0.004** | 4.133 | 0.053 |
| Indole-3-carboxaldehyde | 0.08 | 0.01 | 0.05 | 0.01 | 0.07 | 0.01 | 0.06 | 0.03 | 0.500 | 0.486 | 8.686 | **0.007** | 2.703 | 0.113 |
| Indole | 0.08 | 0.01 | 0.05 | 0.02 | 0.08 | 0.02 | 0.06 | 0.01 | 0.231 | 0.635 | 12.720 | **0.002** | 2.286 | 0.143 |
| Indoxyl Sulfate | 10.56 | 2.66 | 4.01 | 1.43 | 10.08 | 6.07 | 7.22 | 4.80 | 0.975 | 0.334 | 10.280 | **0.004** | 1.706 | 0.203 |
| TMAO | 33.55 | 16.35 | 6.46 | 3.26 | 38.22 | 28.55 | 9.94 | 2.45 | 0.550 | 0.465 | 23.920 | **<0.001** | 0.006 | 0.939 |
| Choline | 36.86 | 6.92 | 32.52 | 5.76 | 33.07 | 4.57 | 30.08 | 4.63 | 2.709 | 0.112 | 2.713 | 0.112 | 0.037 | 0.849 |
| T-a-MCA | 13.61 | 6.11 | 10.82 | 24.80 | 37.19 | 79.22 | 3.88 | 4.07 | 0.397 | 0.534 | 1.745 | 0.199 | 1.088 | 0.307 |
| T-b-MCA | 3.85 | 2.36 | 4.68 | 11.52 | 35.23 | 82.73 | 1.30 | 1.16 | 1.037 | 0.318 | 1.440 | 0.241 | 1.492 | 0.233 |
| TUDCA | 0.93 | 0.29 | 0.67 | 1.07 | 2.85 | 5.77 | 0.41 | 0.18 | 0.749 | 0.395 | 1.929 | 0.177 | 1.171 | 0.289 |
| THDCA | 0.37 | 0.13 | 0.09 | 0.12 | 0.43 | 0.71 | 0.06 | 0.02 | 0.019 | 0.893 | 7.140 | **0.013** | 0.128 | 0.724 |
| TCDCA | 0.10 | 0.05 | 0.07 | 0.12 | 0.52 | 1.02 | 0.04 | 0.01 | 1.237 | 0.277 | 2.178 | 0.153 | 1.653 | 0.210 |
| a-MCA | 2.69 | 0.36 | 1.05 | 0.74 | 1.46 | 0.57 | 2.16 | 2.51 | 0.003 | 0.956 | 0.910 | 0.349 | 5.141 | **0.032** |
| b-MCA | 0.84 | 0.28 | 0.36 | 0.24 | 0.52 | 0.25 | 0.85 | 0.94 | 0.200 | 0.659 | 0.168 | 0.685 | 4.206 | 0.051 |
| CA | 2.27 | 1.09 | 0.31 | 0.30 | 0.71 | 0.41 | 0.99 | 1.18 | 1.852 | 0.186 | 7.005 | **0.014** | 12.440 | **0.002** |
| UDCA | 0.45 | 0.18 | 0.20 | 0.13 | 0.26 | 0.16 | 0.42 | 0.38 | 0.044 | 0.836 | 0.284 | 0.599 | 5.412 | **0.028** |

| HDCA | 0.71 | 0.18 | 0.09 | 0.10 | 0.33 | 0.23 | 0.19 | 0.20 | 4.509 | **0.044** | 30.390 | **<0.001** | 12.570 | **0.002** |
| --- | --- | --- | --- | --- | --- | --- | --- | --- | --- | --- | --- | --- | --- | --- |
| CDCA | 0.30 | 0.18 | 0.17 | 0.08 | 0.18 | 0.16 | 0.12 | 0.04 | 8.414 | **0.008** | 1.998 | 0.170 | 1.942 | 0.176 |
| DCA | 1.34 | 0.44 | 0.15 | 0.12 | 0.80 | 0.65 | 0.34 | 0.22 | 1.420 | 0.245 | 32.580 | **<0.001** | 6.470 | **0.018** |
| LCA | 0.87 | 0.14 | 0.92 | 0.17 | 0.70 | 0.18 | 0.63 | 0.21 | 11.110 | **0.003** | 0.108 | 0.745 | 0.681 | 0.417 |
| TCA | 2.75 | 1.42 | 0.10 | 0.05 | 0.07 | 0.06 | 0.17 | 0.10 | 11.440 | **0.002** | 21.340 | **<0.001** | 17.510 | **0.003** |
| TDCA | 0.31 | 0.33 | 0.02 | 0.01 | 0.01 | 0.00 | 0.03 | 0.02 | 5.654 | **0.025** | 4.738 | **0.039** | 6.521 | **0.017** |


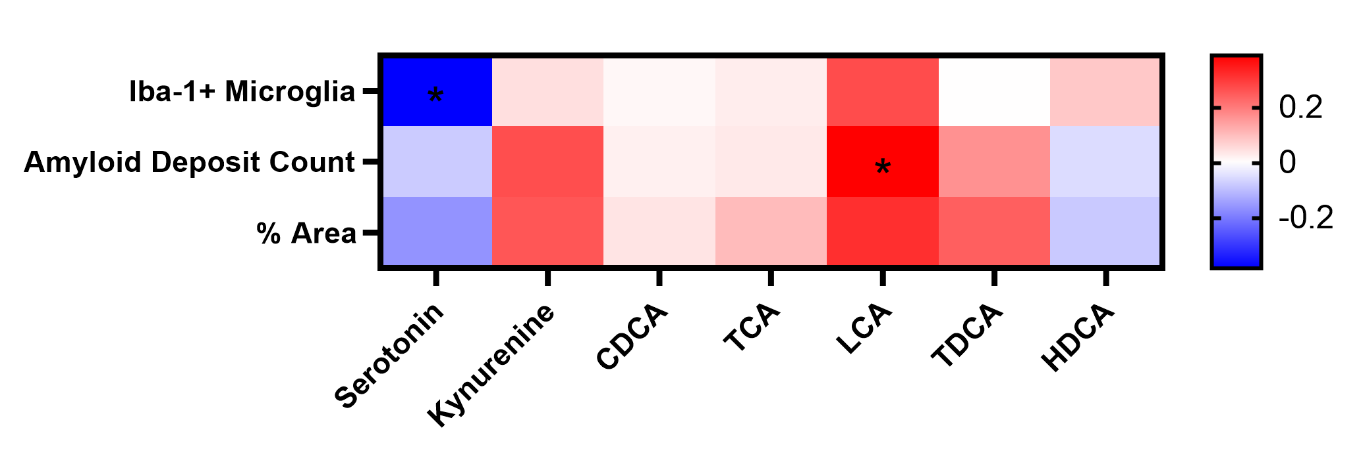


**Supplementary Figure S4: Heatmap displaying the correlation between AD neuropathology and metabolites significantly modulated by the effect of diet (p<0.05).**

**Supplementary Table S5: An effect of diet was observed with amyloid beta-related genes.** Bold values are significant at P_FDR_<0.1

| Gene | Description | Category | log2FoldChange | P-value | FDR P-value |
| --- | --- | --- | --- | --- | --- |
| *Vcam1* | vascular cell adhesion molecule 1 | cellular response to amyloid-beta | 0.51 | <0.001 | **0.027** |
| *Lrp2* | low density lipoprotein receptor-related protein 2 | amyloid-beta clearance | 1.73 | 0.001 | **0.045** |
| *Clu* | clusterin | amyloid-beta binding | 0.85 | 0.000 | **0.051** |
| *Ager* | advanced glycosylation end product-specific receptor | amyloid-beta binding | -1.32 | 0.000 | **0.077** |
| *Fcgr2b* | Fc receptor, IgG, low affinity IIb | amyloid-beta binding | 0.69 | 0.003 | 0.192 |
| *Itga4* | integrin alpha 4 | cellular response to amyloid-beta | 0.66 | 0.005 | 0.235 |
| *Tlr2* | toll-like receptor 2 | amyloid-beta binding | 0.71 | 0.011 | 0.305 |
| *Efna1* | ephrin A1 | positive regulation of amyloid-beta formation (amyloid-beta formation) | 0.41 | 0.013 | 0.329 |
| *Rab11b* | RAB11B, member RAS oncogene family | amyloid-beta clearance by transcytosis | -0.13 | 0.013 | 0.334 |
| *Dlgap3* | DLG associated protein 3 | amyloid-beta binding | -0.50 | 0.017 | 0.357 |
| *Itgb2* | integrin beta 2 | amyloid-beta binding | 0.41 | 0.017 | 0.366 |
| *Lrrtm3* | leucine rich repeat transmembrane neuronal 3 | positive regulation of amyloid-beta formation (amyloid-beta formation) | -0.31 | 0.021 | 0.396 |
| *Ttr* | transthyretin | amyloid-beta binding | 2.35 | 0.022 | 0.400 |
| *Fzd4* | frizzled class receptor 4 | amyloid-beta binding | 0.55 | 0.023 | 0.407 |
| *Pin1* | peptidyl-prolyl cis/trans isomerase, NIMA-interacting 1 | negative regulation of amyloid-beta formation (amyloid-beta formation) | -0.22 | 0.023 | 0.409 |
| *Tgfb2* | transforming growth factor, beta 2 | amyloid-beta binding | 0.59 | 0.024 | 0.410 |
| *Igf1* | insulin-like growth factor 1 | negative regulation of amyloid-beta formation (amyloid-beta formation) | 0.46 | 0.025 | 0.415 |
| *Picalm* | phosphatidylinositol binding clathrin assembly protein | amyloid-beta clearance by transcytosis | 0.16 | 0.028 | 0.438 |
| *Necab3* | N-terminal EF-hand calcium binding protein 3 | amyloid beta (A4) precursor protein-binding, family A, member 2 binding protein | -0.47 | 0.028 | 0.438 |
| *Apoa1* | apolipoprotein A-I | amyloid-beta binding | -1.71 | 0.027 | 0.457 |
| *Cacnb1* | calcium channel, voltage-dependent, beta 1 subunit | cellular response to amyloid-beta | -0.34 | 0.042 | 0.501 |
| *Gja1* | gap junction protein, alpha 1 | cellular response to amyloid-beta | 0.25 | 0.043 | 0.502 |
| *Tlr6* | toll-like receptor 6 | cellular response to amyloid-beta | 0.86 | 0.043 | 0.502 |
| *Grin1* | glutamate receptor, ionotropic, NMDA1 (zeta 1) | amyloid-beta binding | -0.25 | 0.044 | 0.505 |
| *Sp1* | trans-acting transcription factor 1 | positive regulation of amyloid-beta formation (amyloid-beta formation) | 0.29 | 0.045 | 0.512 |
| *Gga3* | golgi associated, gamma adaptin ear containing, ARF binding protein 3 | negative regulation of amyloid-beta formation (amyloid-beta formation) | -0.13 | 0.048 | 0.520 |
| *Aph1a* | aph1 homolog A, gamma secretase subunit | amyloid-beta formation | -0.16 | 0.051 | 0.533 |
| *Ace* | angiotensin I converting enzyme | amyloid-beta metabolic process | 0.52 | 0.059 | 0.567 |
| *Rtn1* | reticulon 1 | negative regulation of amyloid-beta formation (amyloid-beta formation) | -0.22 | 0.063 | 0.577 |
| *Aplp1* | amyloid beta precursor like protein 1 | amyloid beta precursor like protein 1 | -0.18 | 0.071 | 0.592 |
| *Apba3* | amyloid beta precursor protein binding family A member 3 | amyloid beta precursor protein binding family A member 3 | -0.24 | 0.074 | 0.600 |
| *Psenen* | presenilin enhancer gamma secretase subunit | amyloid-beta formation | -0.15 | 0.076 | 0.607 |
| *Snx6* | sorting nexin 6 | cellular response to amyloid-beta | 0.17 | 0.080 | 0.615 |
| *Mme* | membrane metallo endopeptidase | amyloid-beta metabolic process | -0.57 | 0.087 | 0.630 |
| *Chrna7* | cholinergic receptor, nicotinic, alpha polypeptide 7 | amyloid-beta binding | 0.53 | 0.092 | 0.634 |
| *Nae1* | NEDD8 activating enzyme E1 subunit 1 | amyloid beta precursor protein binding protein 1 | 0.18 | 0.095 | 0.638 |
| *Appbp2* | amyloid beta precursor protein binding protein 2 | amyloid beta precursor protein binding protein 2 | 0.13 | 0.096 | 0.640 |
| *Trem2* | triggering receptor expressed on myeloid cells 2 | amyloid-beta binding | 0.37 | 0.102 | 0.653 |
| *Cacna1b* | calcium channel, voltage-dependent, N type, alpha 1B subunit | response to amyloid-beta | -0.12 | 0.140 | 0.710 |
| *Lrpap1* | low density lipoprotein receptor-related protein associated protein 1 | amyloid-beta clearance by transcytosis | -0.10 | 0.142 | 0.713 |
| *Cdk5* | cyclin dependent kinase 5 | cellular response to amyloid-beta | -0.11 | 0.147 | 0.719 |
| *Lgmn* | legumain | cellular response to amyloid-beta | 0.15 | 0.150 | 0.722 |
| *Ldlr* | low density lipoprotein receptor | amyloid-beta binding | 0.14 | 0.153 | 0.724 |
| *Mmp9* | matrix metallopeptidase 9 | response to amyloid-beta | -0.52 | 0.158 | 0.729 |
| *Becn1* | beclin 1, autophagy related | amyloid-beta metabolic process | -0.13 | 0.165 | 0.738 |
| *Pfdn6* | prefoldin subunit 6 | amyloid-beta binding | -0.16 | 0.172 | 0.746 |
| *Gria1* | glutamate receptor, ionotropic, AMPA1 (alpha 1) | amyloid-beta binding | 0.21 | 0.173 | 0.746 |
| *Rtn2* | reticulon 2 (Z-band associated protein) | negative regulation of amyloid-beta formation (amyloid-beta formation) | -0.11 | 0.178 | 0.753 |
| *Insr* | insulin receptor | amyloid-beta binding | 0.12 | 0.186 | 0.761 |
| *Clstn1* | calsyntenin 1 | amyloid-beta binding | -0.20 | 0.188 | 0.763 |
| *Gsk3a* | glycogen synthase kinase 3 alpha | positive regulation of amyloid-beta formation (amyloid-beta formation) | -0.17 | 0.200 | 0.777 |
| *Gria3* | glutamate receptor, ionotropic, AMPA3 (alpha 3) | amyloid-beta binding | -0.19 | 0.221 | 0.793 |
| *Lrp8* | low density lipoprotein receptor-related protein 8, apolipoprotein e receptor | amyloid-beta binding | 0.11 | 0.229 | 0.795 |
| *Unc13a* | unc-13 homolog A | amyloid-beta metabolic process | -0.18 | 0.229 | 0.795 |
| *Slc2a13* | solute carrier family 2 (facilitated glucose transporter), member 13 | positive regulation of amyloid-beta formation (amyloid-beta formation) | -0.14 | 0.248 | 0.811 |
| *Tlr4* | toll-like receptor 4 | cellular response to amyloid-beta | 0.54 | 0.254 | 0.816 |
| *Pfdn5* | prefoldin 5 | amyloid-beta binding | -0.11 | 0.255 | 0.818 |
| *Pfdn4* | prefoldin 4 | amyloid-beta binding | 0.19 | 0.258 | 0.820 |
| *Ephb2* | Eph receptor B2 | amyloid-beta binding | 0.24 | 0.264 | 0.824 |
| *Clstn2* | calsyntenin 2 | amyloid-beta binding | 0.26 | 0.265 | 0.824 |
| *Ldlrap1* | low density lipoprotein receptor adaptor protein 1 | amyloid-beta binding | 0.41 | 0.268 | 0.826 |
| *Csnk1e* | casein kinase 1, epsilon | positive regulation of amyloid-beta formation (amyloid-beta formation) | -0.11 | 0.268 | 0.827 |
| *Pfdn1* | prefoldin 1 | amyloid-beta binding | -0.14 | 0.278 | 0.833 |
| *Sorl1* | sortilin-related receptor, LDLR class A repeats-containing | amyloid-beta binding | 0.19 | 0.305 | 0.849 |
| *Itm2a* | integral membrane protein 2A | amyloid-beta binding | 0.16 | 0.310 | 0.851 |
| *Apbb1* | amyloid beta precursor protein binding family B member 1 | amyloid beta precursor protein binding family B member 1 | -0.15 | 0.310 | 0.852 |
| *Epha4* | Eph receptor A4 | positive regulation of amyloid-beta formation (amyloid-beta formation) | 0.39 | 0.316 | 0.856 |
| *Cd74* | CD74 antigen (invariant polypeptide of major histocompatibility complex, class II antigen-associated) | amyloid-beta binding | 0.61 | 0.324 | 0.859 |
| *Aph1c* | aph1 homolog C, gamma secretase subunit | amyloid-beta formation | 0.15 | 0.329 | 0.863 |
| *Itgam* | integrin alpha M | amyloid-beta clearance | 0.15 | 0.335 | 0.865 |
| *C3* | complement component 3 | amyloid-beta clearance | 0.60 | 0.340 | 0.866 |
| *Cyp51* | cytochrome P450, family 51 | negative regulation of amyloid-beta clearance (amyloid-beta clearance) | 0.09 | 0.350 | 0.870 |
| *Ngfr* | nerve growth factor receptor (TNFR superfamily, member 16) | amyloid-beta binding | -0.43 | 0.357 | 0.870 |
| *Ntrk2* | neurotrophic tyrosine kinase, receptor, type 2 | negative regulation of amyloid-beta formation (amyloid-beta formation) | 0.08 | 0.360 | 0.872 |
| *Itm2c* | integral membrane protein 2C | amyloid-beta binding | -0.09 | 0.373 | 0.877 |
| *Bace2* | beta-site APP-cleaving enzyme 2 | amyloid-beta metabolic process | 0.19 | 0.393 | 0.886 |
| *Srf* | serum response factor | negative regulation of amyloid-beta clearance (amyloid-beta clearance) | -0.16 | 0.400 | 0.890 |
| *Itm2b* | integral membrane protein 2B | amyloid-beta binding | 0.05 | 0.409 | 0.891 |
| *Tmed10* | transmembrane p24 trafficking protein 10 | regulation of amyloid-beta formation (amyloid-beta formation) | 0.07 | 0.416 | 0.893 |
| *Igf1r* | insulin-like growth factor I receptor | amyloid-beta clearance | 0.11 | 0.434 | 0.899 |
| *Rela* | v-rel reticuloendotheliosis viral oncogene homolog A (avian) | positive regulation of amyloid-beta formation (amyloid-beta formation) | 0.08 | 0.436 | 0.900 |
| *Ramp3* | receptor (calcitonin) activity modifying protein 3 | response to amyloid-beta | 0.40 | 0.447 | 0.904 |
| *Gsap* | gamma-secretase activating protein | amyloid-beta binding | 0.12 | 0.473 | 0.912 |
| *Ifngr1* | interferon gamma receptor 1 | negative regulation of amyloid-beta clearance (amyloid-beta clearance) | 0.09 | 0.482 | 0.913 |
| *Apbb2* | amyloid beta precursor protein binding family B member 2 | amyloid beta precursor protein binding family B member 2 | 0.07 | 0.499 | 0.918 |
| *Hap1* | huntingtin-associated protein 1 | negative regulation of amyloid-beta formation (amyloid-beta formation) | -0.19 | 0.504 | 0.920 |
| *Cltc* | clathrin heavy chain | amyloid-beta clearance by transcytosis | 0.08 | 0.506 | 0.920 |
| *Apbb1ip* | amyloid beta precursor protein binding family B member 1 interacting protein | amyloid beta precursor protein binding family B member 1 interacting protein | 0.12 | 0.516 | 0.925 |
| *Pfdn2* | prefoldin 2 | amyloid-beta binding | -0.09 | 0.519 | 0.926 |
| *Abca2* | ATP-binding cassette, sub-family A member 2 | positive regulation of amyloid-beta formation (amyloid-beta formation) | 0.07 | 0.546 | 0.932 |
| *Ide* | insulin degrading enzyme | amyloid-beta binding | 0.09 | 0.547 | 0.932 |
| *Hmgcr* | 3-hydroxy-3-methylglutaryl-Coenzyme A reductase | negative regulation of amyloid-beta clearance (amyloid-beta clearance) | 0.09 | 0.554 | 0.933 |
| *Aph1b* | aph1 homolog B, gamma secretase subunit | amyloid-beta formation | 0.07 | 0.557 | 0.934 |
| *Icam1* | intercellular adhesion molecule 1 | cellular response to amyloid-beta | 0.15 | 0.564 | 0.936 |
| *Il4* | interleukin 4 | positive regulation of amyloid-beta clearance (amyloid-beta clearance) | 0.08 | 0.568 | 0.936 |
| *Bcl2l2* | BCL2-like 2 | cellular response to amyloid-beta | -0.05 | 0.569 | 0.936 |
| *Clstn3* | calsyntenin 3 | amyloid-beta binding | -0.04 | 0.572 | 0.936 |
| *Atp1a3* | ATPase, Na+/K+ transporting, alpha 3 polypeptide | amyloid-beta binding | -0.05 | 0.577 | 0.936 |
| *Col25a1* | collagen, type XXV, alpha 1 | amyloid-beta binding | 0.10 | 0.574 | 0.936 |
| *Cst3* | cystatin C | amyloid-beta binding | -0.06 | 0.575 | 0.936 |
| *Bace1* | beta-site APP cleaving enzyme 1 | amyloid-beta binding | 0.09 | 0.581 | 0.937 |
| *Rtn3* | reticulon 3 | negative regulation of amyloid-beta formation (amyloid-beta formation) | 0.06 | 0.587 | 0.938 |
| *Cacna2d1* | calcium channel, voltage-dependent, alpha2/delta subunit 1 | cellular response to amyloid-beta | -0.08 | 0.589 | 0.938 |
| *Cacna1a* | calcium channel, voltage-dependent, P/Q type, alpha 1A subunit | response to amyloid-beta | -0.07 | 0.622 | 0.944 |
| *Mgat3* | mannoside acetylglucosaminyltransferase 3 | amyloid-beta metabolic process | 0.07 | 0.628 | 0.945 |
| *Gria2* | glutamate receptor, ionotropic, AMPA2 (alpha 2) | amyloid-beta binding | 0.07 | 0.632 | 0.946 |
| *Tm2d1* | TM2 domain containing 1 | amyloid-beta binding | -0.06 | 0.635 | 0.946 |
| *Lrp1* | low density lipoprotein receptor-related protein 1 | amyloid-beta clearance by transcytosis | 0.04 | 0.636 | 0.946 |
| *Scarb1* | scavenger receptor class B, member 1 | amyloid-beta binding | -0.06 | 0.647 | 0.948 |
| *Olfm1* | olfactomedin 1 | amyloid-beta binding | -0.11 | 0.653 | 0.950 |
| *Apoe* | apolipoprotein E | amyloid-beta binding | -0.07 | 0.654 | 0.950 |
| *Abcc1* | ATP-binding cassette, sub-family C member 1 | cellular response to amyloid-beta | -0.04 | 0.701 | 0.961 |
| *Foxo3* | forkhead box O3 | cellular response to amyloid-beta | -0.08 | 0.703 | 0.961 |
| *Hsd17b10* | hydroxysteroid (17-beta) dehydrogenase 10 | amyloid-beta binding | 0.05 | 0.713 | 0.963 |
| *Ttpa* | tocopherol (alpha) transfer protein | positive regulation of amyloid-beta clearance (amyloid-beta clearance) | -0.09 | 0.716 | 0.963 |
| *Grm5* | glutamate receptor, metabotropic 5 | cellular response to amyloid-beta | 0.05 | 0.718 | 0.964 |
| *App* | amyloid beta precursor protein | amyloid beta precursor protein | -0.04 | 0.731 | 0.967 |
| *Parp1* | poly (ADP-ribose) polymerase family, member 1 | cellular response to amyloid-beta | 0.05 | 0.750 | 0.971 |
| *Aplp2* | amyloid beta precursor-like protein 2 | amyloid beta precursor-like protein 2 | 0.03 | 0.761 | 0.972 |
| *Fpr2* | formyl peptide receptor 2 | amyloid-beta binding | 0.27 | 0.753 | 0.972 |
| *Calcr* | calcitonin receptor | amyloid-beta binding | -0.39 | 0.775 | 0.974 |
| *Rab11a* | RAB11A, member RAS oncogene family | amyloid-beta clearance by transcytosis | -0.02 | 0.777 | 0.974 |
| *Dyrk1a* | dual-specificity tyrosine phosphorylation regulated kinase 1a | amyloid-beta formation | -0.03 | 0.782 | 0.975 |
| *Adam10* | a disintegrin and metallopeptidase domain 10 | amyloid-beta formation | 0.04 | 0.784 | 0.975 |
| *Psen1* | presenilin 1 | amyloid-beta formation | 0.02 | 0.794 | 0.976 |
| *Apeh* | acylpeptide hydrolase | amyloid-beta metabolic process | 0.06 | 0.794 | 0.976 |
| *Apba1* | amyloid beta precursor protein binding family A member 1 | amyloid beta precursor protein binding family A member 1 | 0.03 | 0.797 | 0.977 |
| *Apba2* | amyloid beta precursor protein binding family A member 2 | amyloid beta precursor protein binding family A member 2 | 0.04 | 0.800 | 0.978 |
| *Prnp* | prion protein | amyloid-beta binding | -0.03 | 0.807 | 0.979 |
| *Psen2* | presenilin 2 | amyloid-beta formation | -0.03 | 0.806 | 0.979 |
| *Casp3* | caspase 3 | positive regulation of amyloid-beta formation (amyloid-beta formation) | 0.05 | 0.810 | 0.980 |
| *Abcg1* | ATP binding cassette subfamily G member 1 | positive regulation of amyloid-beta formation (amyloid-beta formation) | 0.03 | 0.811 | 0.981 |
| *Rock1* | Rho-associated coiled-coil containing protein kinase 1 | amyloid-beta complex | 0.03 | 0.811 | 0.981 |
| *Vbp1* | von Hippel-Lindau binding protein 1 | amyloid-beta binding | 0.03 | 0.815 | 0.982 |
| *Cryab* | crystallin, alpha B | amyloid-beta binding | 0.04 | 0.821 | 0.982 |
| *Pla2g3* | phospholipase A2, group III | negative regulation of amyloid-beta clearance (amyloid-beta clearance) | 0.06 | 0.851 | 0.986 |
| *Rtn4* | reticulon 4 | negative regulation of amyloid-beta formation (amyloid-beta formation) | -0.02 | 0.854 | 0.986 |
| *Ldlrad3* | low density lipoprotein receptor class A domain containing 3 | amyloid-beta binding | 0.03 | 0.857 | 0.987 |
| *Hba-a1* | hemoglobin alpha, adult chain 1 | amyloid-beta binding | 0.15 | 0.874 | 0.989 |
| *Gprasp2* | G protein-coupled receptor associated sorting protein 2 | amyloid-beta binding | 0.04 | 0.876 | 0.989 |
| *Bin1* | bridging integrator 1 | negative regulation of amyloid-beta formation (amyloid-beta formation) | 0.01 | 0.877 | 0.990 |
| *Adrb2* | adrenergic receptor, beta 2 | amyloid-beta binding | 0.04 | 0.888 | 0.992 |
| *Nat8f1* | N-acetyltransferase 8 (GCN5-related) family member 1 | amyloid-beta metabolic process | -0.02 | 0.903 | 0.992 |
| *Rock2* | Rho-associated coiled-coil containing protein kinase 2 | positive regulation of amyloid-beta formation (amyloid-beta formation) | 0.02 | 0.903 | 0.992 |
| *Fbxo2* | F-box protein 2 | amyloid-beta binding | -0.01 | 0.920 | 0.992 |
| *Lrp4* | low density lipoprotein receptor-related protein 4 | amyloid-beta clearance by cellular catabolic process | -0.02 | 0.922 | 0.993 |
| *Abca7* | ATP-binding cassette, sub-family A member 7 | amyloid-beta clearance by cellular catabolic process | -0.01 | 0.937 | 0.994 |
| *Sirt1* | sirtuin 1 | cellular response to amyloid-beta | 0.01 | 0.938 | 0.994 |
| *Rab5a* | RAB5A, member RAS oncogene family | amyloid-beta clearance by transcytosis | 0.01 | 0.944 | 0.995 |
| *Fzd5* | frizzled class receptor 5 | amyloid-beta binding | -0.03 | 0.947 | 0.995 |
| *Spon1* | spondin 1, (f-spondin) extracellular matrix protein | negative regulation of amyloid-beta formation (amyloid-beta formation) | 0.00 | 0.961 | 0.997 |
| *Fyn* | Fyn proto-oncogene | response to amyloid-beta | 0.00 | 0.983 | 0.999 |
| *Gsk3b* | glycogen synthase kinase 3 beta | cellular response to amyloid-beta | 0.00 | 0.981 | 0.999 |
| *Ncstn* | nicastrin | amyloid-beta formation | 0.00 | 0.977 | 0.999 |

**Supplementary Table S6: Amyloid beta-related genes are not significantly modulated by the main effect of sex.**

| Gene | Description | Category | log2FoldChange | P-value | FDR P-value |
| --- | --- | --- | --- | --- | --- |
| *Cryab* | crystallin, alpha B | amyloid-beta binding | 0.64 | <0.001 | 0.247 |
| *Itgb2* | integrin beta 2 | amyloid-beta binding | 0.58 | 0.001 | 0.373 |
| *Vbp1* | von Hippel-Lindau binding protein 1 | amyloid-beta binding | 0.32 | 0.003 | 0.607 |
| *Trem2* | triggering receptor expressed on myeloid cells 2 | amyloid-beta binding | 0.65 | 0.004 | 0.670 |
| *Apbb1ip* | amyloid beta precursor protein binding family B member 1 interacting protein | amyloid beta precursor protein binding family B member 1 interacting protein | 0.55 | 0.005 | 0.692 |
| *C3* | complement component 3 | amyloid-beta clearance | 1.91 | 0.005 | 0.692 |
| *Atp1a3* | ATPase, Na+/K+ transporting, alpha 3 polypeptide | amyloid-beta binding | -0.20 | 0.016 | 0.840 |
| *Lgmn* | legumain | cellular response to amyloid-beta | 0.26 | 0.017 | 0.840 |
| *Fcgr2b* | Fc receptor, IgG, low affinity IIb | amyloid-beta binding | 0.58 | 0.017 | 0.841 |
| *Itgam* | integrin alpha M | amyloid-beta clearance | 0.38 | 0.022 | 0.888 |
| *Scarb1* | scavenger receptor class B, member 1 | amyloid-beta binding | -0.27 | 0.043 | 0.905 |
| *Rtn2* | reticulon 2 (Z-band associated protein) | negative regulation of amyloid-beta formation (amyloid-beta formation) | -0.16 | 0.048 | 0.907 |
| *Adrb2* | adrenergic receptor, beta 2 | amyloid-beta binding | 0.54 | 0.067 | 0.909 |
| *Pla2g3* | phospholipase A2, group III | negative regulation of amyloid-beta clearance (amyloid-beta clearance) | -0.58 | 0.055 | 0.909 |
| *Rab11a* | RAB11A, member RAS oncogene family | amyloid-beta clearance by transcytosis | 0.15 | 0.058 | 0.909 |
| *Mgat3* | mannoside acetylglucosaminyltransferase 3 | amyloid-beta metabolic process | -0.26 | 0.075 | 0.921 |
| *Psenen* | presenilin enhancer gamma secretase subunit | amyloid-beta formation | 0.15 | 0.079 | 0.923 |
| *Chrna7* | cholinergic receptor, nicotinic, alpha polypeptide 7 | amyloid-beta binding | -0.54 | 0.091 | 0.938 |
| *Abcg1* | ATP binding cassette subfamily G member 1 | positive regulation of amyloid-beta formation (amyloid-beta formation) | -0.17 | 0.150 | 0.946 |
| *Apoe* | apolipoprotein E | amyloid-beta binding | 0.21 | 0.191 | 0.946 |
| *Bace2* | beta-site APP-cleaving enzyme 2 | amyloid-beta metabolic process | -0.33 | 0.146 | 0.946 |
| *Bin1* | bridging integrator 1 | negative regulation of amyloid-beta formation (amyloid-beta formation) | 0.13 | 0.166 | 0.946 |
| *Calcr* | calcitonin receptor | amyloid-beta binding | 1.84 | 0.176 | 0.946 |
| *Cdk5* | cyclin dependent kinase 5 | cellular response to amyloid-beta | 0.12 | 0.112 | 0.946 |
| *Cst3* | cystatin C | amyloid-beta binding | 0.15 | 0.141 | 0.946 |
| *Gga3* | golgi associated, gamma adaptin ear containing, ARF binding protein 3 | negative regulation of amyloid-beta formation (amyloid-beta formation) | -0.09 | 0.172 | 0.946 |
| *Gria1* | glutamate receptor, ionotropic, AMPA1 (alpha 1) | amyloid-beta binding | -0.21 | 0.169 | 0.946 |
| *Gria2* | glutamate receptor, ionotropic, AMPA2 (alpha 2) | amyloid-beta binding | -0.20 | 0.169 | 0.946 |
| *Grm5* | glutamate receptor, metabotropic 5 | cellular response to amyloid-beta | -0.22 | 0.131 | 0.946 |
| *Gsap* | gamma-secretase activating protein | amyloid-beta binding | -0.22 | 0.167 | 0.946 |
| *Gsk3b* | glycogen synthase kinase 3 beta | cellular response to amyloid-beta | -0.16 | 0.145 | 0.946 |
| *Il4* | interleukin 4 | positive regulation of amyloid-beta clearance (amyloid-beta clearance) | 0.20 | 0.190 | 0.946 |
| *Mme* | membrane metallo endopeptidase | amyloid-beta metabolic process | -0.45 | 0.175 | 0.946 |
| *Parp1* | poly (ADP-ribose) polymerase family, member 1 | cellular response to amyloid-beta | -0.26 | 0.115 | 0.946 |
| *Psen2* | presenilin 2 | amyloid-beta formation | 0.17 | 0.187 | 0.946 |
| *Tm2d1* | TM2 domain containing 1 | amyloid-beta binding | 0.16 | 0.188 | 0.946 |
| *Ttr* | transthyretin | amyloid-beta binding | 1.40 | 0.173 | 0.946 |
| *Grin1* | glutamate receptor, ionotropic, NMDA1 (zeta 1) | amyloid-beta binding | -0.16 | 0.207 | 0.948 |
| *Fyn* | Fyn proto-oncogene | response to amyloid-beta | -0.12 | 0.216 | 0.953 |
| *Apbb2* | amyloid beta precursor protein binding family B member 2 | amyloid beta precursor protein binding family B member 2 | 0.12 | 0.248 | 0.957 |
| *Cltc* | clathrin heavy chain | amyloid-beta clearance by transcytosis | -0.14 | 0.237 | 0.957 |
| *Itm2c* | integral membrane protein 2C | amyloid-beta binding | -0.11 | 0.253 | 0.957 |
| *Pfdn1* | prefoldin 1 | amyloid-beta binding | 0.15 | 0.268 | 0.957 |
| *Srf* | serum response factor | negative regulation of amyloid-beta clearance (amyloid-beta clearance) | -0.23 | 0.235 | 0.957 |
| *Tlr2* | toll-like receptor 2 | amyloid-beta binding | 0.33 | 0.264 | 0.957 |
| *Foxo3* | forkhead box O3 | cellular response to amyloid-beta | -0.21 | 0.296 | 0.963 |
| *Gsk3a* | glycogen synthase kinase 3 alpha | positive regulation of amyloid-beta formation (amyloid-beta formation) | -0.14 | 0.297 | 0.963 |
| *Cacnb1* | calcium channel, voltage-dependent, beta 1 subunit | cellular response to amyloid-beta | -0.17 | 0.300 | 0.963 |
| *Dyrk1a* | dual-specificity tyrosine phosphorylation regulated kinase 1a | amyloid-beta formation | -0.10 | 0.305 | 0.963 |
| *Itm2a* | integral membrane protein 2A | amyloid-beta binding | -0.16 | 0.312 | 0.963 |
| *Ldlrad3* | low density lipoprotein receptor class A domain containing 3 | amyloid-beta binding | 0.18 | 0.309 | 0.963 |
| *Slc2a13* | solute carrier family 2 (facilitated glucose transporter), member 13 | positive regulation of amyloid-beta formation (amyloid-beta formation) | -0.12 | 0.314 | 0.963 |
| *Unc13a* | unc-13 homolog A | amyloid-beta metabolic process | -0.15 | 0.311 | 0.963 |
| *Pfdn5* | prefoldin 5 | amyloid-beta binding | 0.10 | 0.323 | 0.966 |
| *Gprasp2* | G protein-coupled receptor associated sorting protein 2 | amyloid-beta binding | 0.26 | 0.325 | 0.967 |
| *Dlgap3* | DLG associated protein 3 | amyloid-beta binding | -0.20 | 0.337 | 0.967 |
| *Lrp8* | low density lipoprotein receptor-related protein 8, apolipoprotein e receptor | amyloid-beta binding | -0.09 | 0.336 | 0.967 |
| *Ramp3* | receptor (calcitonin) activity modifying protein 3 | response to amyloid-beta | 0.51 | 0.343 | 0.969 |
| *Snx6* | sorting nexin 6 | cellular response to amyloid-beta | 0.09 | 0.348 | 0.969 |
| *Sorl1* | sortilin-related receptor, LDLR class A repeats-containing | amyloid-beta binding | -0.17 | 0.341 | 0.969 |
| *Tmed10* | transmembrane p24 trafficking protein 10 | regulation of amyloid-beta formation (amyloid-beta formation) | 0.08 | 0.346 | 0.969 |
| *Cacna1a* | calcium channel, voltage-dependent, P/Q type, alpha 1A subunit | response to amyloid-beta | -0.12 | 0.363 | 0.969 |
| *Insr* | insulin receptor | amyloid-beta binding | -0.08 | 0.368 | 0.970 |
| *Cacna1b* | calcium channel, voltage-dependent, N type, alpha 1B subunit | response to amyloid-beta | 0.07 | 0.374 | 0.971 |
| *Ngfr* | nerve growth factor receptor (TNFR superfamily, member 16) | amyloid-beta binding | 0.42 | 0.372 | 0.971 |
| *Tgfb2* | transforming growth factor, beta 2 | amyloid-beta binding | -0.23 | 0.371 | 0.971 |
| *Prnp* | prion protein | amyloid-beta binding | 0.10 | 0.377 | 0.972 |
| *Abcc1* | ATP-binding cassette, sub-family C member 1 | cellular response to amyloid-beta | -0.08 | 0.378 | 0.972 |
| *Sirt1* | sirtuin 1 | cellular response to amyloid-beta | -0.13 | 0.384 | 0.973 |
| *Ifngr1* | interferon gamma receptor 1 | negative regulation of amyloid-beta clearance (amyloid-beta clearance) | 0.12 | 0.387 | 0.974 |
| *Hmgcr* | 3-hydroxy-3-methylglutaryl-Coenzyme A reductase | negative regulation of amyloid-beta clearance (amyloid-beta clearance) | -0.13 | 0.402 | 0.974 |
| *Icam1* | intercellular adhesion molecule 1 | cellular response to amyloid-beta | 0.22 | 0.421 | 0.974 |
| *Lrp2* | low density lipoprotein receptor-related protein 2 | amyloid-beta clearance | 0.52 | 0.427 | 0.974 |
| *Lrp4* | low density lipoprotein receptor-related protein 4 | amyloid-beta clearance by cellular catabolic process | -0.13 | 0.430 | 0.974 |
| *Pin1* | peptidyl-prolyl cis/trans isomerase, NIMA-interacting 1 | negative regulation of amyloid-beta formation (amyloid-beta formation) | -0.08 | 0.398 | 0.974 |
| *Rela* | v-rel reticuloendotheliosis viral oncogene homolog A (avian) | positive regulation of amyloid-beta formation (amyloid-beta formation) | -0.08 | 0.429 | 0.974 |
| *Rtn4* | reticulon 4 | negative regulation of amyloid-beta formation (amyloid-beta formation) | -0.11 | 0.417 | 0.974 |
| *Tlr4* | toll-like receptor 4 | cellular response to amyloid-beta | 0.40 | 0.425 | 0.974 |
| *Ide* | insulin degrading enzyme | amyloid-beta binding | -0.12 | 0.433 | 0.975 |
| *Cd74* | CD74 antigen (invariant polypeptide of major histocompatibility complex, class II antigen-associated) | amyloid-beta binding | 0.48 | 0.440 | 0.975 |
| *Lrrtm3* | leucine rich repeat transmembrane neuronal 3 | positive regulation of amyloid-beta formation (amyloid-beta formation) | -0.10 | 0.461 | 0.979 |
| *Ldlr* | low density lipoprotein receptor | amyloid-beta binding | 0.07 | 0.466 | 0.980 |
| *Rab11b* | RAB11B, member RAS oncogene family | amyloid-beta clearance by transcytosis | 0.04 | 0.469 | 0.980 |
| *Aph1c* | aph1 homolog C, gamma secretase subunit | amyloid-beta formation | 0.11 | 0.475 | 0.981 |
| *Epha4* | Eph receptor A4 | positive regulation of amyloid-beta formation (amyloid-beta formation) | -0.27 | 0.479 | 0.981 |
| *Apba1* | amyloid beta precursor protein binding family A member 1 | amyloid beta precursor protein binding family A member 1 | -0.07 | 0.521 | 0.983 |
| *Apba3* | amyloid beta precursor protein binding family A member 3 | amyloid beta precursor protein binding family A member 3 | 0.09 | 0.516 | 0.983 |
| *Hap1* | huntingtin-associated protein 1 | negative regulation of amyloid-beta formation (amyloid-beta formation) | 0.19 | 0.515 | 0.983 |
| *Spon1* | spondin 1, (f-spondin) extracellular matrix protein | negative regulation of amyloid-beta formation (amyloid-beta formation) | 0.06 | 0.529 | 0.983 |
| *Bcl2l2* | BCL2-like 2 | cellular response to amyloid-beta | -0.06 | 0.542 | 0.983 |
| *Cyp51* | cytochrome P450, family 51 | negative regulation of amyloid-beta clearance (amyloid-beta clearance) | 0.06 | 0.546 | 0.984 |
| *Efna1* | ephrin A1 | positive regulation of amyloid-beta formation (amyloid-beta formation) | 0.10 | 0.569 | 0.987 |
| *Rtn1* | reticulon 1 | negative regulation of amyloid-beta formation (amyloid-beta formation) | 0.07 | 0.564 | 0.987 |
| *Clstn1* | calsyntenin 1 | amyloid-beta binding | -0.08 | 0.584 | 0.987 |
| *Psen1* | presenilin 1 | amyloid-beta formation | -0.05 | 0.582 | 0.987 |
| *Tlr6* | toll-like receptor 6 | cellular response to amyloid-beta | -0.25 | 0.575 | 0.987 |
| *Fzd4* | frizzled class receptor 4 | amyloid-beta binding | -0.13 | 0.589 | 0.990 |
| *Ntrk2* | neurotrophic tyrosine kinase, receptor, type 2 | negative regulation of amyloid-beta formation (amyloid-beta formation) | -0.05 | 0.598 | 0.992 |
| *Picalm* | phosphatidylinositol binding clathrin assembly protein | amyloid-beta clearance by transcytosis | 0.04 | 0.599 | 0.992 |
| *Ace* | angiotensin I converting enzyme | amyloid-beta metabolic process | 0.14 | 0.612 | 0.994 |
| *Casp3* | caspase 3 | positive regulation of amyloid-beta formation (amyloid-beta formation) | -0.09 | 0.647 | 0.994 |
| *Col25a1* | collagen, type XXV, alpha 1 | amyloid-beta binding | 0.08 | 0.643 | 0.994 |
| *Gja1* | gap junction protein, alpha 1 | cellular response to amyloid-beta | -0.05 | 0.670 | 0.994 |
| *Gria3* | glutamate receptor, ionotropic, AMPA3 (alpha 3) | amyloid-beta binding | -0.08 | 0.619 | 0.994 |
| *Hba-a1* | hemoglobin alpha, adult chain 1 | amyloid-beta binding | 0.40 | 0.672 | 0.994 |
| *Lrp1* | low density lipoprotein receptor-related protein 1 | amyloid-beta clearance by transcytosis | -0.03 | 0.672 | 0.994 |
| *Nae1* | NEDD8 activating enzyme E1 subunit 1 | amyloid beta precursor protein binding protein 1 | -0.05 | 0.627 | 0.994 |
| *Pfdn2* | prefoldin 2 | amyloid-beta binding | 0.07 | 0.630 | 0.994 |
| *Pfdn4* | prefoldin 4 | amyloid-beta binding | 0.08 | 0.651 | 0.994 |
| *Sp1* | trans-acting transcription factor 1 | positive regulation of amyloid-beta formation (amyloid-beta formation) | -0.06 | 0.663 | 0.994 |
| *Vcam1* | vascular cell adhesion molecule 1 | cellular response to amyloid-beta | 0.06 | 0.661 | 0.994 |
| *App* | amyloid beta precursor protein | amyloid beta precursor protein | -0.05 | 0.685 | 0.995 |
| *Rock2* | Rho-associated coiled-coil containing protein kinase 2 | positive regulation of amyloid-beta formation (amyloid-beta formation) | -0.06 | 0.691 | 0.996 |
| *Ttpa* | tocopherol (alpha) transfer protein | positive regulation of amyloid-beta clearance (amyloid-beta clearance) | 0.10 | 0.696 | 0.996 |
| *Clu* | clusterin | amyloid-beta binding | -0.09 | 0.701 | 0.996 |
| *Hsd17b10* | hydroxysteroid (17-beta) dehydrogenase 10 | amyloid-beta binding | -0.05 | 0.708 | 0.997 |
| *Itm2b* | integral membrane protein 2B | amyloid-beta binding | 0.02 | 0.715 | 0.998 |
| *Nat8f1* | N-acetyltransferase 8 (GCN5-related) family member 1 | amyloid-beta metabolic process | 0.06 | 0.726 | 0.998 |
| *Aplp2* | amyloid beta precursor-like protein 2 | amyloid beta precursor-like protein 2 | -0.03 | 0.728 | 0.998 |
| *Aplp1* | amyloid beta precursor like protein 1 | amyloid beta precursor like protein 1 | 0.03 | 0.732 | 0.999 |
| *Fbxo2* | F-box protein 2 | amyloid-beta binding | 0.04 | 0.732 | 0.999 |
| *Abca7* | ATP-binding cassette, sub-family A member 7 | amyloid-beta clearance by cellular catabolic process | 0.04 | 0.761 | 0.999 |
| *Appbp2* | amyloid beta precursor protein binding protein 2 | amyloid beta precursor protein binding protein 2 | -0.02 | 0.770 | 0.999 |
| *Bace1* | beta-site APP cleaving enzyme 1 | amyloid-beta binding | -0.05 | 0.760 | 0.999 |
| *Ldlrap1* | low density lipoprotein receptor adaptor protein 1 | amyloid-beta binding | 0.12 | 0.755 | 0.999 |
| *Mmp9* | matrix metallopeptidase 9 | response to amyloid-beta | 0.12 | 0.739 | 0.999 |
| *Abca2* | ATP-binding cassette, sub-family A member 2 | positive regulation of amyloid-beta formation (amyloid-beta formation) | 0.03 | 0.789 | 0.999 |
| *Itga4* | integrin alpha 4 | cellular response to amyloid-beta | -0.06 | 0.805 | 0.999 |
| *Igf1r* | insulin-like growth factor I receptor | amyloid-beta clearance | -0.03 | 0.809 | 1.000 |
| *Adam10* | a disintegrin and metallopeptidase domain 10 | amyloid-beta formation | 0.02 | 0.876 | 1.000 |
| *Ager* | advanced glycosylation end product-specific receptor | amyloid-beta binding | 0.01 | 0.972 | 1.000 |
| *Apba2* | amyloid beta precursor protein binding family A member 2 | amyloid beta precursor protein binding family A member 2 | -0.03 | 0.841 | 1.000 |
| *Apbb1* | amyloid beta precursor protein binding family B member 1 | amyloid beta precursor protein binding family B member 1 | -0.01 | 0.967 | 1.000 |
| *Apeh* | acylpeptide hydrolase | amyloid-beta metabolic process | 0.01 | 0.956 | 1.000 |
| *Aph1a* | aph1 homolog A, gamma secretase subunit | amyloid-beta formation | 0.01 | 0.903 | 1.000 |
| *Aph1b* | aph1 homolog B, gamma secretase subunit | amyloid-beta formation | -0.03 | 0.839 | 1.000 |
| *Apoa1* | apolipoprotein A-I | amyloid-beta binding | 0.05 | 0.936 | 1.000 |
| *Becn1* | beclin 1, autophagy related | amyloid-beta metabolic process | -0.01 | 0.901 | 1.000 |
| *Cacna2d1* | calcium channel, voltage-dependent, alpha2/delta subunit 1 | cellular response to amyloid-beta | 0.01 | 0.938 | 1.000 |
| *Clstn2* | calsyntenin 2 | amyloid-beta binding | -0.02 | 0.914 | 1.000 |
| *Clstn3* | calsyntenin 3 | amyloid-beta binding | -0.01 | 0.908 | 1.000 |
| *Csnk1e* | casein kinase 1, epsilon | positive regulation of amyloid-beta formation (amyloid-beta formation) | 0.01 | 0.911 | 1.000 |
| *Ephb2* | Eph receptor B2 | amyloid-beta binding | -0.05 | 0.827 | 1.000 |
| *Fpr2* | formyl peptide receptor 2 | amyloid-beta binding | 0.01 | 0.993 | 1.000 |
| *Fzd5* | frizzled class receptor 5 | amyloid-beta binding | -0.06 | 0.892 | 1.000 |
| *Igf1* | insulin-like growth factor 1 | negative regulation of amyloid-beta formation (amyloid-beta formation) | 0.02 | 0.932 | 1.000 |
| *Lrpap1* | low density lipoprotein receptor-related protein associated protein 1 | amyloid-beta clearance by transcytosis | 0.01 | 0.910 | 1.000 |
| *Ncstn* | nicastrin | amyloid-beta formation | -0.01 | 0.905 | 1.000 |
| *Necab3* | N-terminal EF-hand calcium binding protein 3 | amyloid beta (A4) precursor protein-binding, family A, member 2 binding protein | -0.00 | 0.986 | 1.000 |
| *Olfm1* | olfactomedin 1 | amyloid-beta binding | 0.04 | 0.888 | 1.000 |
| *Pfdn6* | prefoldin subunit 6 | amyloid-beta binding | -0.00 | 0.995 | 1.000 |
| *Rab5a* | RAB5A, member RAS oncogene family | amyloid-beta clearance by transcytosis | 0.01 | 0.888 | 1.000 |
| *Rock1* | Rho-associated coiled-coil containing protein kinase 1 | amyloid-beta complex | -0.01 | 0.913 | 1.000 |
| *Rtn3* | reticulon 3 | negative regulation of amyloid-beta formation (amyloid-beta formation) | 0.00 | 0.996 | 1.000 |


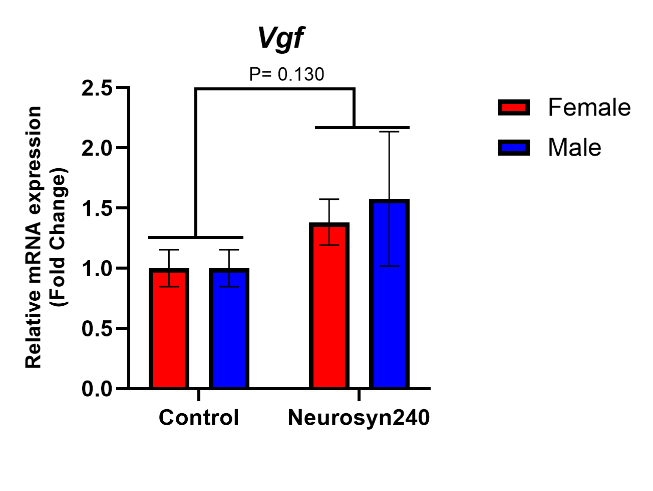

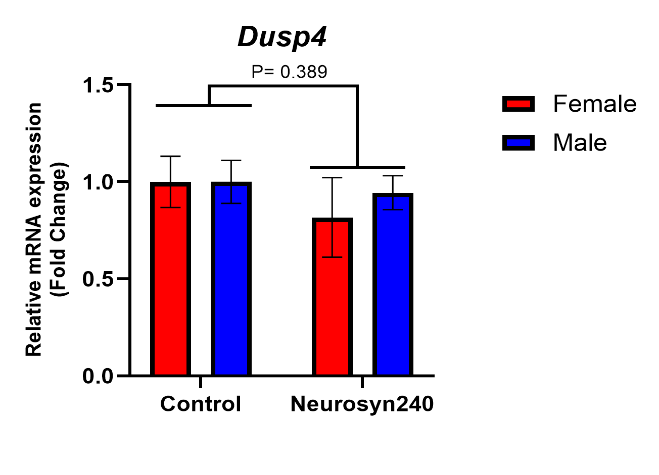

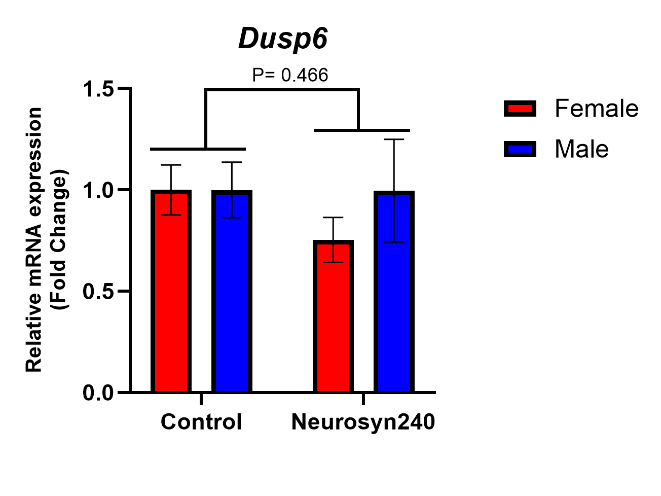


**Supplementary Figure S5: Neurosyn240 does not significantly alter hippocampal expression of *Vgf* (A), *Dusp4* (B), or *Dusp6* (C)**

C

A

B


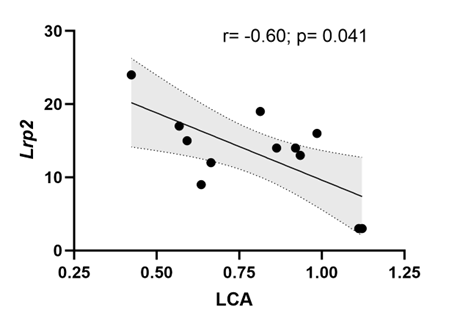


**Supplementary Figure S6: LCA concentrations correlate with hippocampal *Lrp2.***
